# Genome-Wide Identification of the MIKC-Type MADS-Box Gene Family in *Gossypium hirsutum* L. Unravels Their Roles in Flowering

**DOI:** 10.3389/fpls.2017.00384

**Published:** 2017-03-22

**Authors:** Zhongying Ren, Daoqian Yu, Zhaoen Yang, Changfeng Li, Ghulam Qanmber, Yi Li, Jie Li, Zhao Liu, Lili Lu, Lingling Wang, Hua Zhang, Quanjia Chen, Fuguang Li, Zuoren Yang

**Affiliations:** ^1^Xinjiang Research Base, State Key Laboratory of Cotton Biology, Xinjiang Agriculture UniversityUrumqi, China; ^2^Institute of Cotton Research, Chinese Academy of Agricultural SciencesAnyang, China; ^3^Cotton Research Institute, Anhui Academy of Agricultural SciencesHefei, China

**Keywords:** *Gossypium hirsutum* L., *GhMIKCs*, phylogeny, structure, expression patterns, flower

## Abstract

Cotton is one of the major world oil crops. Cottonseed oil meets the increasing demand of fried food, ruminant feed, and renewable bio-fuels. MADS intervening keratin-like and C-terminal (MIKC)-type MADS-box genes encode transcription factors that have crucial roles in various plant developmental processes. Nevertheless, this gene family has not been characterized, nor its functions investigated, in cotton. Here, we performed a comprehensive analysis of MIKC-type MADS genes in the tetraploid *Gossypium hirsutum* L., which is the most widely cultivated cotton species. In total, 110 *GhMIKC* genes were identified and phylogenetically classified into 13 subfamilies. The Flowering locus C (*FLC*) subfamily was absent in the *Gossypium hirsutum* L. genome but is found in Arabidopsis and *Vitis vinifera* L. Among the genes, 108 were distributed across the 13 A and 12 of the D genome's chromosomes, while two were located in scaffolds. *GhMIKCs* within subfamilies displayed similar exon/intron characteristics and conserved motif compositions. According to RNA-sequencing, most MIKC genes exhibited high flowering-associated expression profiles. A quantitative real-time PCR analysis revealed that some crucial MIKC genes determined the identities of the five flower organs. Furthermore, the overexpression of *GhAGL17.9* in Arabidopsis caused an early flowering phenotype. Meanwhile, the expression levels of the flowering-related genes *CONSTANS (CO), LEAFY (LFY)* and *SUPPRESSOR OF OVEREXPRESSION OF CONSTANS1 (SOC1)* were significantly increased in these lines. These results provide useful information for future studies of *GhMIKCs'* regulation of cotton flowering.

## Introduction

Transcription factors play an indispensable role in growth and development, and MADS transcription factor family members have been detected in the genomes of plants, animals, and fungi (Becker et al., 2000; Becker and Theissen, 2003; Messenguy and Dubois, 2003). In monophyletic evolution, they are divided into two classes: type I and type II (Alvarez-Buylla et al., 2000). The type I MADS-box genes are serum response factor-like genes in animals and fungi, while they are M-type genes in plants. They are characterized by a highly conserved MADS domain of the 58–60 amino acids, located in the N-terminal region of the proteins, which are involved in DNA binding and dimerization. Functional investigations have been mainly restricted to Arabidopsis (Parenicová et al., 2003). The type II family plays a significant role in regulating flowering during plant development (Mondragon-Palomino, 2013). Type II genes are closely related to the myocyte enhancer factor-2-like genes of animals and yeast. However, MADS intervening keratin-like and C-terminal (MIKC)-type MADS-box genes are found only in plants.

The MIKC-type plant genes contain three additional domains other than the MADS (M): Intervening (I), Keratin (K), and the C-terminal (C) domains (Theißen et al., 1996; Kaufmann et al., 2005). The I domain forms DNA-binding dimers, which is less conserved (Riechmann et al., 1996). The K domain, which consists of ~70 amino acids, is mainly responsible for dimerization by a coiled-coil structure (Ma et al., 1991; Fan et al., 1997). The C domain exhibits transactivation and mediates protein–protein interactions (Kramer and Irish, 1999; Honma and Goto, 2001). Based on structure divergence at the I domain, the MIKC-type genes are classified into two subgroups, MIKC^C^ and MIKC^*^. Earlier investigations found 39 and 37 MIKC^C^ genes in Arabidopsis and *O. sativa*, respectively (Parenicová et al., 2003; Arora et al., 2007). The MIKC^C^ type plays a crucial role in the flowering time, floral organ identity determination and fruit ripening in plant growth and development (Theissen, 2001; Becker and Theissen, 2003; Theissen and Melzer, 2007; Li et al., 2016).

The genetic floral organ model is derived from the analysis of homeotic floral mutants. The ABC model was named after three classes of genes (A, B, and C) (Coen and Meyerowitz, 1991), and has developed into the more exact ABCDE model. MIKC^C^ family genes have combined and determined the identities of the floral organs: sepals (A + E), petals (A + B + E), stamens (B + C + E), carpels (C + E), and ovules (D + E) (Bowman et al., 1991; Coen and Meyerowitz, 1991; Ma and Depamphilis, 2000; Zahn et al., 2006; Silva et al., 2015). In Arabidopsis, the functional genes were divided into five classes: Class A: *APETALA1* (*AP1*); Class B: *PISTILATA (PI)* and *AP3*; Class C: *AGAMOUS (AG*) (Acri-Nunes-Miranda and Mondragón-Palomino, 2014); Class D: *SEEDSTICK/AGAMOUS-LIKE11 (STK/AGL11)*; and Class E: *SEPALLATA (SEP1, SEP2, SEP3*, and *SEP4*) (Ferrándiz et al., 2000; Pinyopich et al., 2003). Other MIKC^C^ genes that regulated flowering time and flower initiation have been identified as follows: *Suppressor of Overexpression Of Constans1 (SOC1)* (Lee et al., 2000; Hepworth et al., 2002); *Flowering Locus c (FLC)* (Michaels and Amasino, 1999; Searle et al., 2006; Reeves et al., 2007); *AGAMOUSLIKE GENE 24 (AGL24)* (Michaels et al., 2003; Liu et al., 2008) and *Short Vegetative Phase (SVP)* (Hartmann et al., 2000; Michaels et al., 2003; Lee et al., 2007). Others are involved in fruit ripening, such as *SHATTERPROOF 1–2* and *FUL* (Ferrándiz et al., 2000; Liljegren et al., 2000), in seed pigmentation and endothelium development, such as *TRANSPARENT TESTA16* (Nesi et al., 2002), and in root development such as *AGL12* and *AGL17* (Rounsley et al., 1995; Tapia-López et al., 2008). Studies of the evolutionary history of MIKC genes have explored the internal mechanisms behind their functional diversification in plant growth and development.

Cotton is not only the most important source of natural fiber for textile industry (Pang et al., 2010), but also a major contributor in world oilseed economy. The extracted cottonseed oil has long been considered to be a good vegetable oil (Michaelk et al., 2010; Sawan, 2014; Zhang et al., 2014). Simultaneously, as an alternative and sustainable oil source, cottonseed oil has been developed into biodiesel and used as substitutes for petroleum (Carlsson, 2009; Alhassan et al., 2014). As the top five oil crops in the world (Wang et al., 2016), cottonseed oil occupies about 21% of the cottonseed production (Malik and Ahsan, 2016; Wang et al., 2016; Yang and Zheng, 2016). The formation of cotton seed originates from ovule which is an important part of floral organs. *G. hirsutum*'s MIKC functions are highly significant in plant developmental processes. Especially, a number of genes could involve in the development of flower morphology (Honma and Goto, 2001; Messenguy and Dubois, 2003). For example, *GhMADS3*, a homolog of Arabidopsis *AG* and putative C function gene, overexpression can improve sepal-to-carpel and petal-to-stamen transformations in transgenic tobacco (Guo et al., 2007). *GhMADS13*, a high homolog of Arabidopsis *AGL6*, overexpression significantly promotes flower buds in cotton (Wu et al., 2009), and *GhMADS14* is enhanced gradually during the early stages of fiber elongation (Zhou et al., 2014). In previous study, 53 members of the *G. hirsutum* MIKC^C^ gene family were identified based on the *G. raimondii* genome (Jiang et al., 2014). However, owing to the lack of *G. hirsutum* genome sequences, a comprehensive analysis of MIKC-type MADS genes in *G. hirsutum* has not yet been reported.

Recently, the *G. hirsutum* genome was sequenced. To systematically analyze the MIKC-Type MADS family genes in *G. hirsutum*, 92 MIKC^*C*^, and 18 MIKC^*^ members of the MIKC family were identified from the whole *G. hirsutum* genome. Phylogeny, structures, locations and expression patterns were comprehensively analyzed. AGL17 is the biggest subgroup, and the involvement of *GhAGL17* subfamily gene in regulating flowering was confirmed by ectopic expression in Arabidopsis. Our findings provide a foundation for the genetic improvement of cotton flowering.

## Materials and methods

### Identification of MIKC genes in *Gossypium hirsutum* L

To identify members of the MIKC gene family in *G. hirsutum*, Arabidopsis MIKC sequences were obtained from the TAIR database (http://www.arabidopsis.org) and used as queries for a BLASTP algorithm-based against the *G. hirsutum* genome database (https://www.cottongen.org/species/Gossypium_hirsutum/nbi-AD1_genome_v1.1) (Zhang et al., 2015). The MIKC protein domain was analyzed using the Hidden Markov Model (HMM) from the Pfam database (http://pfam.xfam.org/). The SRF-TF and K-box domains were confirmed by Pfam accessions (PF00319 and PF01486, respectively). All of the candidate proteins were manually checked using the above described methods to remove the redundant sequences.

### Phylogenetic tree construction

To construct a MIKC-protein phylogenetic tree using MEGA 6.06, MIKC proteins from four plant species, Arabidopsis, *O. sativa, V. vinifera*, and *G. hirsutum*, were employed. The neighbor-joining method with amino acid p-distance was applied to construct the tree (Tamura et al., 2011), and the reliability was obtained by bootstrapping with 1,000 replicates.

### Exon/intron structure, motif and chromosomal location analyses

The exon/intron structures of MIKC genes were retrieved by the alignment of predicted coding sequences with corresponding genomic sequences using the gene structure display server (GSDS) program (http://gsds.cbi.pku.edu.cn/).

The online program MEME (http://meme-suite.org/) was employed to determine the conserved motifs in GhMIKCs with the following optimum parameters: a motif width of 8–200 amino acids and a maximum of 13 motifs. The identified motifs were annotated using the program InterProScan (Quevillon et al., 2005).

The chromosomal distributions of MIKC genes were obtained based on genome annotation data. The MapInspect software was applied to draw images of their physical locations in *G. hirsutum*.

### Gene expression analysis

The expression of MIKC family genes were measured using RNA-sequencing method. The raw RNA-sequencing data of *G. hirsutum* TM-1 seven different tissues (root, stem, leaf, flower, ovule, seed, and fiber) was downloaded from the NCBI Gene Expression repository under the accession number PRJNA248163 (Table S4) (https://www.ncbi.nlm.nih.gov/bioproject/PRJNA248163/). The relative data were normalized to calculate the expression levels. Hierarchical clustering was performed using Genesis 1.7.7 (Sturn et al., 2002).

### RNA isolation and the qRT-PCR analysis

*Gossypium hirsutum* L. (cv CCRI24) was cultivated in the field in Zhengzhou, China. Five different tissue parts of flower: sepal, petal, stamen, carpel, and ovule were sampled, respectively at full bloom stage. Arabidopsis (Columbia-0) was used as wild type; the leaves of wild type and transgenic lines grown for 25 days were harvested. All samples were frozen immediately in liquid nitrogen and kept at −80°C for total RNA extraction. Total RNA was extracted from each sample using the TRIzol reagent (TIANGEN, Beijing, China) and treated with RNase-free DNase I. Gel electrophoresis and a Nanodrop2000 nucleic acid analyzer were employed to detect the quality of RNA. The first cDNA strand was synthesized from 1 μg total RNA using the Transcriptor First Strand cDNA Synthesis Kit version DRR047A (TaKaRa, Dalian, China). The cDNA was diluted five times for the next experiments.

The gene-specific primers used for qRT-PCR were listed in Supplementary Table S2 and S3. The *G. hirsutum His3* gene and Arabidopsis *Actin2* gene were used as an internal control respectively. The qRT-PCR was performed using SYBR Green (Roche) on a LightCycler480 system (Roche). Each reaction was conducted in a 96-well plate with a volume of 20 μl. The PCR cycling parameters were as follows: 95°C for 5 min, 40 cycles of 95°C for 10 s, 60°C for 10 s, and 72°C for 10 s, followed by an increase from 60 to 95°C. The relative expression levels were analyzed using the LightCycler® 480 gene scanning software. Three biological replicates were measured and each biological replicate was run three times.

### Isolation of *GhAGL17.9* and transformation of Arabidopsis

We amplified *GhAGL17.9* using cDNA templates from the mix of CCRI24 root, stem, leaf and flower. The amplified product was cloned into vector *pCambia2301* (CAMBIA) containing the *CAULIFLOWER MOSAIC VIRUS* (CaMV) *35S* constitutive promoter, and then, the constructed vector was introduced into *Agrobacterium tumefaciens* GV3101 (Clough and Bent, 1998). Floral dip method was used for Agrobacterium-mediated transformation of Arabidopsis. Positive transgenic lines were selected on MS medium containing kanamycin. To grow the transgenic lines, seedlings were sown in plastic pots filled with a nutrient soil and vermiculite mix. Then, they were grown in a culturing room at 22°C under a 16-h light/8-h dark cycle for 1 month.

## Results

### Identification of MIKC genes in *Gossypium hirsutum* L

**Figure 1:**
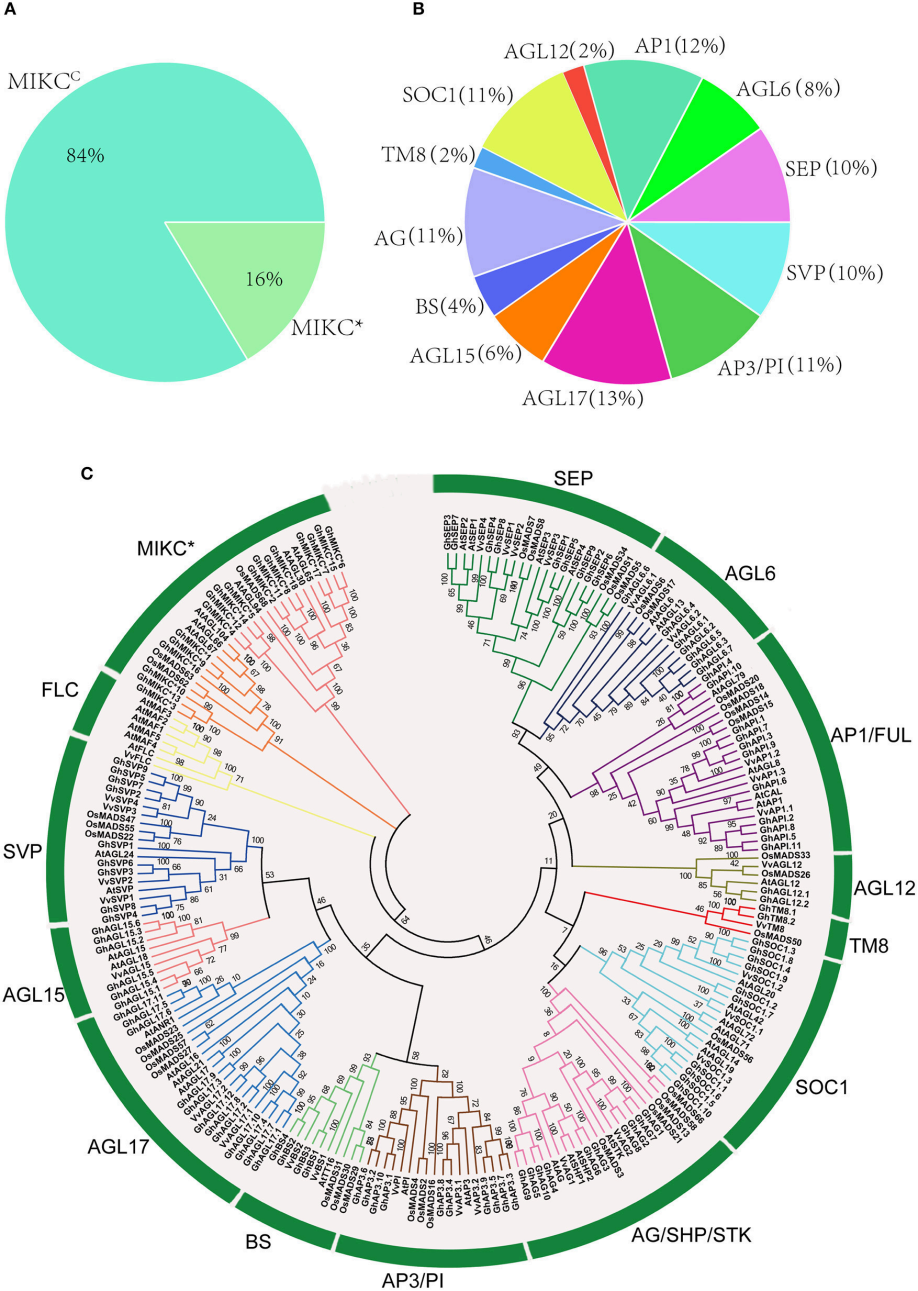
**(A,B)**. Classification and percentage of *GhMIKC* genes. **(C)**. Neighbor-joining phylogenetic tree using MIKC proteins of *Gossypium hirsutum* L., Arabidopsis, *Oryza sativa* L. and *Vitis vinifera* L. Full-length protein sequences were aligned using the MEGA 6.06 program with 1,000 bootstrap replicates. The numbers of the MIKC proteins are listed in Supplementary S1.

HMMER and BLASTP algorithm-based searches were used to identify MIKC protein HMM profiles based on the highly conserved MADS and K-box domains. To identify the maximum number of MIKC genes in *G. hirsutum*, HMMs of SRF-TF, and K-box domains (PF00319 and PF01486, respectively) were extracted from Pfam database to use as queries against protein sequences from the *G. hirsutum* genome (https://www.cottongen.org/species/Gossypium_hirsutum/nbi-AD1_genome_v1.1). A total of 145 putative MIKC proteins were identified. To verify the results, we conducted a multiple sequence alignment and removed 35 redundant sequences. Finally, 110 MIKC protein sequences were identified by confirming their conserved domains using the Pfam web server (Figure S3). From the sequences, 92 MIKC^*C*^ genes and 18 MIKC^*^ genes were identified. Thus, 84% of the MIKC genes were MIKC^C^ in *G. hirsutum* (Figure 1A). The identified MIKC genes were listed with their corresponding locus tag (Table 1). We named the MIKC^*C*^ genes on the basis of their assignment to the 13 previously classified Arabidopsis, *O. sativa* and *P. tremula* subfamilies (Parenicová et al., 2003; Leseberg et al., 2006; Arora et al., 2007). Subgroup AGL17 had the greatest (13%) number of *GhMIKC*^C^ genes; however, subgroups TM8 and AGL12 had the lowest (2%) number of *GhMIKC*^C^ genes (Figure 1B). The *GhMIKC*s' encoding amino acids were relatively conserved, and MIKC^*C*^ proteins were highly conserved, ranging from 200 to 300 amino acids in most cases. MIKC^*^ proteins generally possessed more than 300 amino acids. The chromosomal locations of the 108 *GhMIKCs* were distributed in different subgroups of the A and D genomes, while *GhAGL17.12* and *GhAP3.10* were located on scaffolds.

**Table 1:** ***MIKC* genes identified in *Gossypium hirsutum* L**.

**Gene name**	**Locus ID**	**Arabidopsis ortholog/locus**	**ORF length**	**Introns**	**Chro**	**Chromosome location**	
GhSVP1	Gh_A01G1089	AT2G22540	142	3	A01	41156433	41165546
GhAP3.1	Gh_A01G1608	AT5G20240	252	7	A01	93811588	93817184
GhAP3.2	Gh_A02G0736	AT5G20240	212	6	A02	13128453	13131418
GhAP3.3	Gh_A02G1617	AT3G54340	181	5	A02	82656247	82659133
GhAGL15.1	Gh_A02G1782	AT5G13790	151	3	A02	36467	38800
GhAPI.1	Gh_A03G0634	AT5G60910	231	7	A03	17989506	17995387
GhSEP1	Gh_A03G1085	AT1G24260	243	7	A03	78292489	78296031
GhSVP2	Gh_A03G1551	AT2G22540	211	6	A03	96584815	96588356
GhAGL17.1	Gh_A03G1563	AT2G14210	235	6	A03	96795941	96813515
GhSOC1.1	Gh_A03G2004	AT4G22950	209	6	A03	123532	129632
GhBS1	Gh_A04G0934	AT5G23260	237	5	A04	58360668	58362528
GhAPI.2	Gh_A04G1264	AT1G69120	208	5	A04	62683429	62686813
GhSEP2	Gh_A04G1265	AT2G03710	240	7	A04	62706600	62709888
GhAG1	Gh_A05G2136	AT4G09960	223	7	A05	24318090	24321470
GhAP3.4	Gh_A05G2191	AT3G54340	225	6	A05	25250408	25253113
GhAG2	Gh_A05G2334	AT4G09960	224	7	A05	28279032	28282673
GhAG3	Gh_A05G3267	AT2G42830	234	6	A05	85617722	85626860
GhAGL17.2	Gh_A06G0244	AT3G57230	287	9	A06	3003556	2987706
GhSVP3	Gh_A06G1875	AT2G22540	222	7	A06	12525	16115
GhAPI.3	Gh_A07G0605	AT5G60910	241	7	A07	8448050	8466601
GhAPI.4	Gh_A07G0722	AT1G26310	237	7	A07	11122961	11127898
GhAGL6.1	Gh_A07G1339	AT2G45650	279	8	A07	33028973	33034276
GhSEP3	Gh_A07G1615	AT3G02310	244	7	A07	63573195	63577955
GhAGL6.2	Gh_A08G1148	AT2G45650	246	7	A08	80735208	80752525
GhAGL15.2	Gh_A08G1275	AT5G13790	251	6	A08	84295318	84298231
GhSEP4	Gh_A09G2157	AT3G02310	247	7	A09	74603850	74608301
GhAG4	Gh_A10G2220	AT4G18960	267	7	A10	5463	9549
GhAG5	Gh_A10G2221	AT4G18960	246	6	A10	15954	26053
GhSOC1.2	Gh_A11G0077	AT5G62165	198	6	A11	733666	740007
GhTM8.1	Gh_A11G0343	AT2G45650	236	6	A11	3157934	3160687
GhAGL17.3	Gh_A11G0462	AT4G37940	194	6	A11	4463913	4467818
GhAGL6.3	Gh_A11G0754	AT2G45650	243	7	A11	7460283	7463442
GhSOC1.3	Gh_A11G0755	AT2G45660	219	6	A11	7469708	7466793
GhAGL17.4	Gh_A12G0150	AT4G37940	235	6	A12	2190001	2216459
GhAP3.5	Gh_A12G0570	AT3G54340	224	6	A12	14074225	14075827
GhSVP4	Gh_A12G0775	AT2G22540	220	6	A12	43064710	43067081
GhAGL15.3	Gh_A12G0910	AT5G13790	254	7	A12	59195226	59199007
GhSOC1.4	Gh_A12G0936	AT2G45660	221	6	A12	59611866	59616247
GhSOC1.5	Gh_A12G2048	AT4G22950	240	7	A12	83409840	83379376
GhAGL17.5	Gh_A13G0423	AT3G57230	241	6	A13	5975824	6005673
GhSVP5	Gh_A13G0442	AT2G22540	210	6	A13	6384500	6389623
GhBS2	Gh_A13G0524	AT5G23260	234	5	A13	12156955	12159494
GhAPI.5	Gh_A13G0751	AT1G69120	245	7	A13	28430363	28435475
GhAGL12.1	Gh_A13G0981	AT1G71692	197	6	A13	54345900	54358603
GhAP3.6	Gh_D02G0779	AT5G20240	267	4	D02	12433523	12436663
GhAPI.6	Gh_D02G1311	AT1G69120	220	6	D02	43248417	43251158
GhSEP5	Gh_D02G1502	AT1G24260	243	7	D02	51916080	51919792
GhAGL17.7	Gh_D02G2012	AT3G57230	235	6	D02	64384346	64401650
GhAP3.7	Gh_D03G0105	AT3G54340	224	6	D03	776473	779332
GhAGL15.4	Gh_D03G0626	AT5G13790	151	3	D03	16902671	16900320
GhAPI.7	Gh_D03G0922	AT5G60910	229	7	D03	31494711	31500162
GhSOC1.6	Gh_D03G1493	AT4G22950	209	6	D03	44044063	44050384
GhAG6	Gh_D04G0341	AT2G42830	234	6	D04	5195072	5203980
GhBS3	Gh_D04G1451	AT5G23260	237	5	D04	46216303	46218157
GhAPI.8	Gh_D04G1891	AT1G69120	208	5	D04	51250784	51254164
GhSEP6	Gh_D04G1892	AT2G03710	239	7	D04	51278196	51281401
GhAG7	Gh_D05G2375	AT4G09960	249	6	D05	23662023	23665217
GhAP3.8	Gh_D05G2452	AT3G54340	225	6	D05	24620795	24622467
GhAG8	Gh_D05G2596	AT4G09960	224	7	D05	26719974	26723647
GhAGL17.8	Gh_D06G0245	AT3G57230	278	7	D06	2585610	2602790
GhSVP6	Gh_D06G0267	AT2G22540	222	7	D06	2967937	2971772
GhAPI.9	Gh_D07G0671	AT5G60910	249	7	D07	7915954	7933806
GhAPI.10	Gh_D07G0780	AT5G60910	237	7	D07	9802387	9807245
GhAGL6.4	Gh_D07G1448	AT2G45650	205	6	D07	24514728	24517915
GhSEP7	Gh_D07G1814	AT3G02310	258	7	D07	43509836	43514333
GhAGL6.5	Gh_D08G1430	AT2G45650	246	7	D08	47171916	47179378
GhAGL6.6	Gh_D09G0390	AT2G45650	241	7	D09	14291576	14276476
GhSEP8	Gh_D09G2362	AT3G02310	246	7	D09	50587466	50591473
GhAG9	Gh_D10G0308	AT4G18960	270	6	D10	2675977	2685321
GhAG10	Gh_D10G0309	AT4G18960	267	6	D10	2691566	2695530
GhTM8.2	Gh_D11G0400	AT2G42830	209	7	D11	3355660	3359530
GhAGL17.9	Gh_D11G0534	AT3G57230	217	6	D11	4716974	4720878
GhSOC1.7	Gh_D11G0082	AT5G62165	198	6	D11	761555	756004
GhAGL6.7	Gh_D11G0882	AT2G45650	243	7	D11	7626649	7629794
GhSOC1.8	Gh_D11G0883	AT2G45660	226	6	D11	7646051	7640905
GhAGL15.5	Gh_D11G3150	AT3G57390	253	7	D11	64012119	64007167
GhSVP7	Gh_D12G0156	AT2G22540	217	6	D12	1988003	1993542
GhAGL17.10	Gh_D12G0163	AT4G37940	235	6	D12	2078670	2093157
GhAP3.9	Gh_D12G0585	AT3G54340	371	7	D12	10852076	10878409
GhSVP8	Gh_D12G0778	AT2G22540	220	6	D12	21444908	21447279
GhAGL15.6	Gh_D12G1000	AT5G13790	257	7	D12	35612730	35616501
GhAGL17.11	Gh_D13G0472	AT3G57230	239	6	D13	5578532	5572141
GhSVP9	Gh_D13G0489	AT2G22540	215	6	D13	5951231	5956667
GhBS4	Gh_D13G0605	AT4G18960	223	11	D13	8417153	8434999
GhSEP9	Gh_D13G0877	AT2G03710	244	7	D13	16474813	16478208
GhAPI.11	Gh_D13G0878	AT1G69120	248	7	D13	16637933	16642915
GhAGL12.2	Gh_D13G1226	AT1G71692	197	6	D13	37196257	37208865
GhAGL17.12	Gh_Sca004768G07	AT3G57230	304	7		77172	100229
GhAP3.10	Gh_Sca007246G01	AT5G20240	252	7		1411	8231
GhMIKC^*^1	Gh_A02G0780	AT1G22130	328	9	A02	15645054	15648072
GhMIKC^*^2	Gh_A03G0884	AT2G03060	353	9	A03	56790026	56793057
GhMIKC^*^3	Gh_A05G1797	AT1G22130	308	9	A05	18899832	18897846
GhMIKC^*^4	Gh_A05G2981	AT1G69540	192	7	A05	73507812	73509545
GhMIKC^*^5	Gh_A06G0748	AT1G18750	188	6	A06	25502870	25501592
GhMIKC^*^6	Gh_A07G0593	AT1G18750	380	7	A07	8207711	8211325
GhMIKC^*^7	Gh_A12G1618	AT1G18750	377	10	A12	77294489	77290589
GhMIKC^*^8	Gh_A13G0671	AT1G69540	353	8	A13	20300605	20303927
GhMIKC^*^9	Gh_D02G0829	AT1G77980	336	10	D02	14113941	14116904
GhMIKC^*^10	Gh_D02G0895	AT1G22130	319	8	D02	17340192	17341980
GhMIKC^*^11	Gh_D02G1268	AT2G03060	357	9	D02	41714661	41717693
GhMIKC^*^12	Gh_D04G0771	AT1G69540	192	7	D04	15890346	15900308
GhMIKC^*^13	Gh_D05G1992	AT1G22130	310	9	D05	18333572	18331617
GhMIKC^*^14	Gh_D06G0878	AT1G18750	188	6	D06	16346541	16347795
GhMIKC^*^15	Gh_D07G0660	AT1G18750	358	9	D07	7708262	7712331
GhMIKC^*^16	Gh_D11G2216	AT1G77950	329	9	D11	37092959	37090156
GhMIKC^*^17	Gh_D12G1758	AT1G18750	352	11	D12	49950909	49947011
GhMIKC^*^18	Gh_D13G0785	AT1G69540	399	8	D13	13393230	13397325

### Phylogenetic analysis of the MIKC gene family

To examine the phylogenetic relationships among *G. hirsutum* MIKC proteins and to categorize them within the established subfamilies from other plants, we performed a multiple alignment analysis using the neighbor-joining method of 110 full-length MIKC proteins from *G. hirsutum*, 44 MIKC proteins from *V. vinifera*, 46 MIKC proteins from Arabidopsis, and 41 MIKC proteins from *O. sativa* (Table S1). The MIKC^C^ proteins were divided into 13 subfamilies (SVP, BS, AGL17, AGL15, AP3-PI, AGL12, SOC1, AG/SHP/STK, AP1/FUL, AGL6, SEP, TM8, and FLC; Figure 1C). The AGL17 subgroup was the largest, and the FLC subgroup was absent in the *G. hirsutum* genome. Additionally, no TM8 family members were found in Arabidopsis. TM8 constituted the smallest clade, having only four members, including two GhMIKCs, GhTM8.1, and GhTM8.2. The MIKC^*^ proteins were divided into two subfamilies.

### Gene structure and protein motif analysis

**Figure 2:**
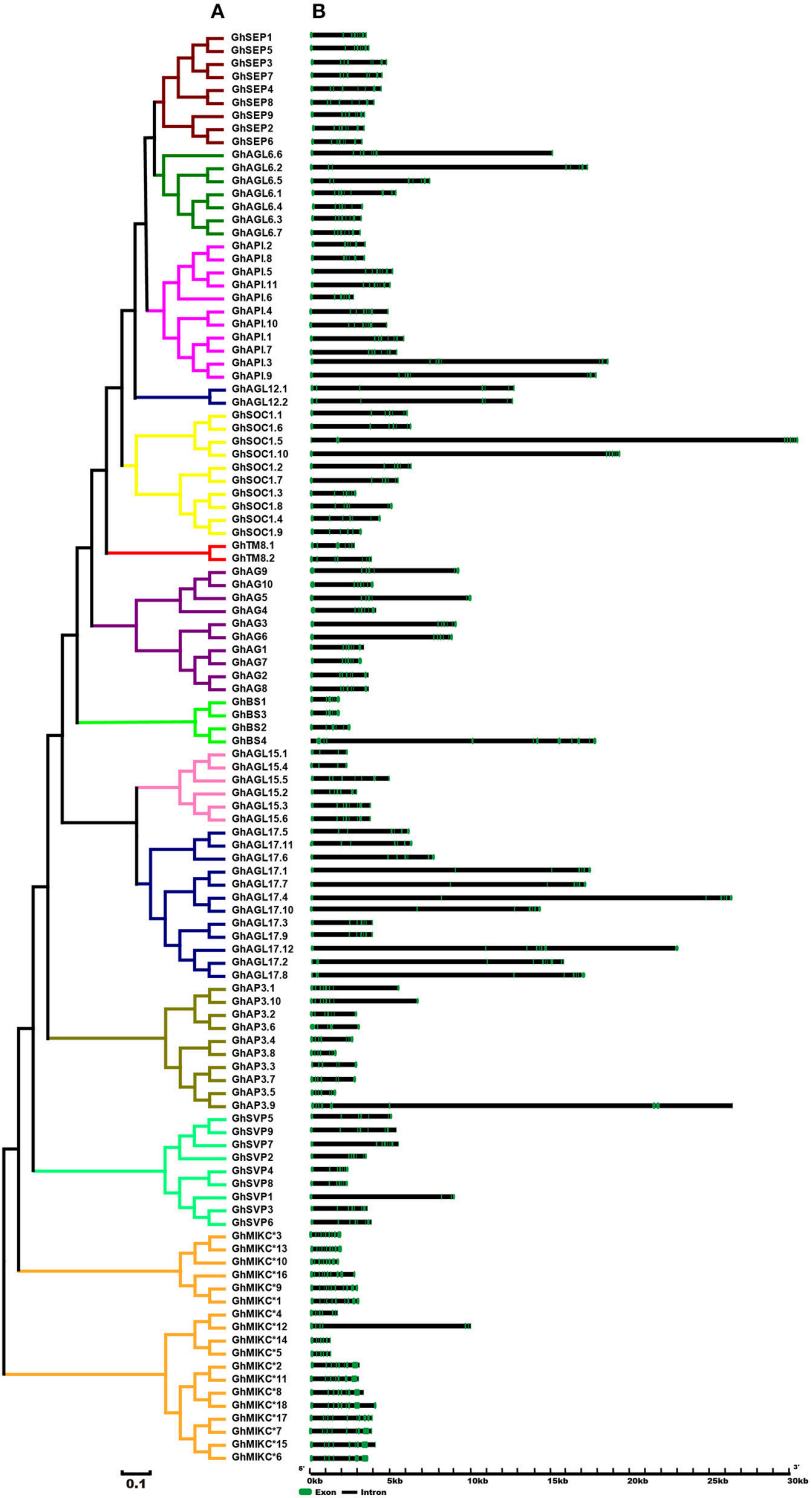
**(A)**. Phylogenetic relationships and **(B)**. Gene structure of MIKC genes in *Gossypium hirsutum* L. The neighbor-joining tree was constructed with MEGA v6.06. The 13 subfamilies are marked with different colored lines. Exons and introns are represented by green and black lines.

A phylogenetic analysis revealed that our tree corresponded to those reported recently in *V. vinifera* and *C. sativus* (Díaz-Riquelme et al., 2009; Hu and Liu, 2012). The structures of the MIKC genes also helped to determine phylogenetic relationships (Figure 2). Most members had significant sequence identities in the same subfamily and similar exon-intron structures, indicating close evolutionary relationships. The most important differences were in the exon-intron lengths (Figure 2B). In general, most members contained eight exons in the *SEP, AGL6*, and *AP1* gene families (except *GhAGL6.1, GhAGL6.4, GhAP1.2, GhAP1.6*, and *GhAPI.8*). The *SVP* (other than *SVP1*) and *AGL12* subgroups had seven exons, whereas *GhAGL15.1* and *GhAGL15.4* of the AGL15 subgroup had four exons, which was consistent with *GhSVP1* of the *SVP* subgroup. The *AGL17* genes displayed relatively longer lengths compared with other subgroup genes. Additionally, *GhBS4* had 11 introns and the first exon was meaningfully shorter, while in *GhSOC1.5*, the second of seven introns was longer than the others. The MIKC^*^ had much shorter gene lengths and more introns than the MIKC^C^. GhMIKC^*^12 had the fourth longest intron, which distinguished it from other members of the MIKC^*^ family.

**Figure 3:**
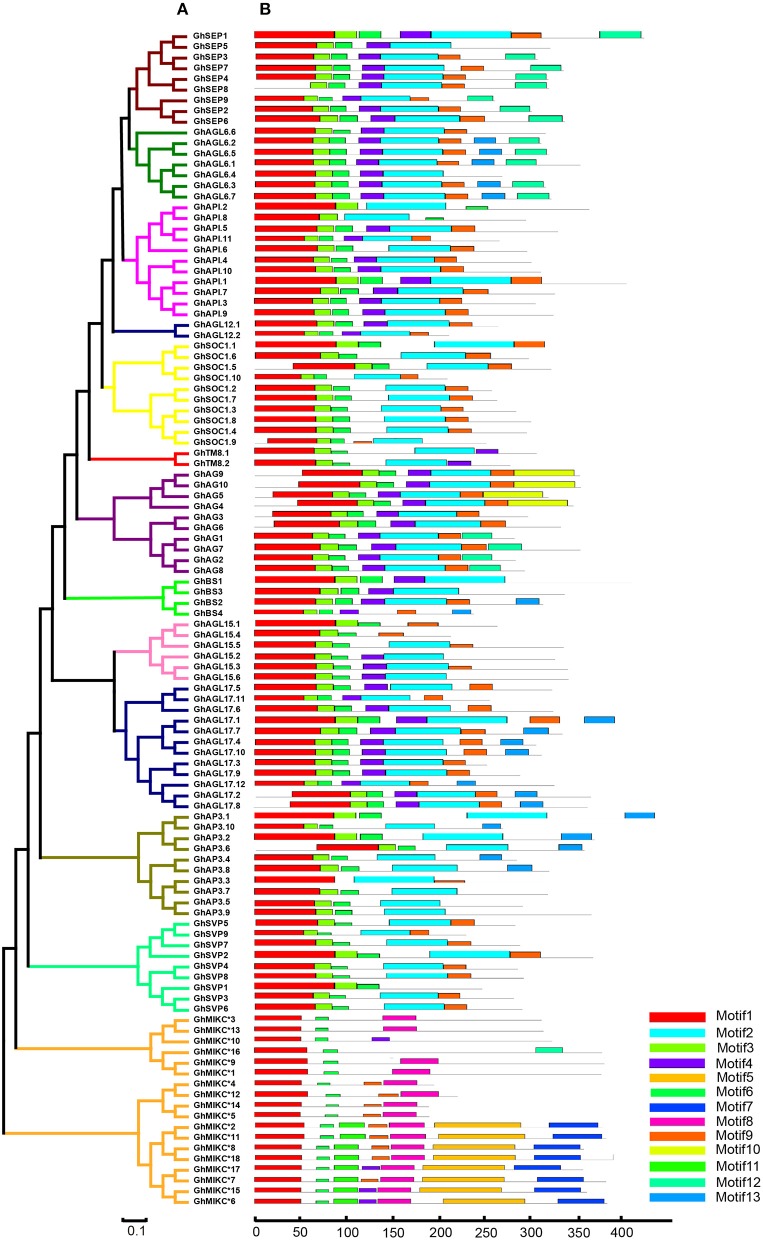
**(A)**. Phylogenetic relationships and **(B)**. Conserved motifs of GhMIKC proteins. The motif compositions were determined using MEME. Motif 1 contains MADS domain, Motifs 2, 4, 5, 8, and 9 contain K-box domains.

We used MEME to analyze MIKC proteins, and 13 conserved motifs were identified (Figure 3B). Most of the closely related MIKC proteins had similar motif type distributions in the same subfamily (Figure 3A). The most striking divergence among the subgroups was in the composition of the C-terminal domains. Motif 1 contained the MADS domain in all of the MIKC families, except GhSEP8. The highly conserved sequence logs were showed in Figure S2. The differences between I regions and K-box domains were distinctly shown in the MIKC^*C*^ and MIKC^*^ proteins (Figure 3B). The K-box domain contained three motifs, 2, 4, and 9, in GhMIKC^C^. However, motif 4, 5, 8, and 9 were present in the GhMIKC^*^ K-box domain, depending on the lengths. The I region in the MIKC^C^ subfamily contained Motifs 3 and 6, while members of the MIKC^*^ contained motifs 6 and 11, which resulted in a longer I region.

### Chromosomes distributions of *GhMIKC* genes

**Figure 4:**
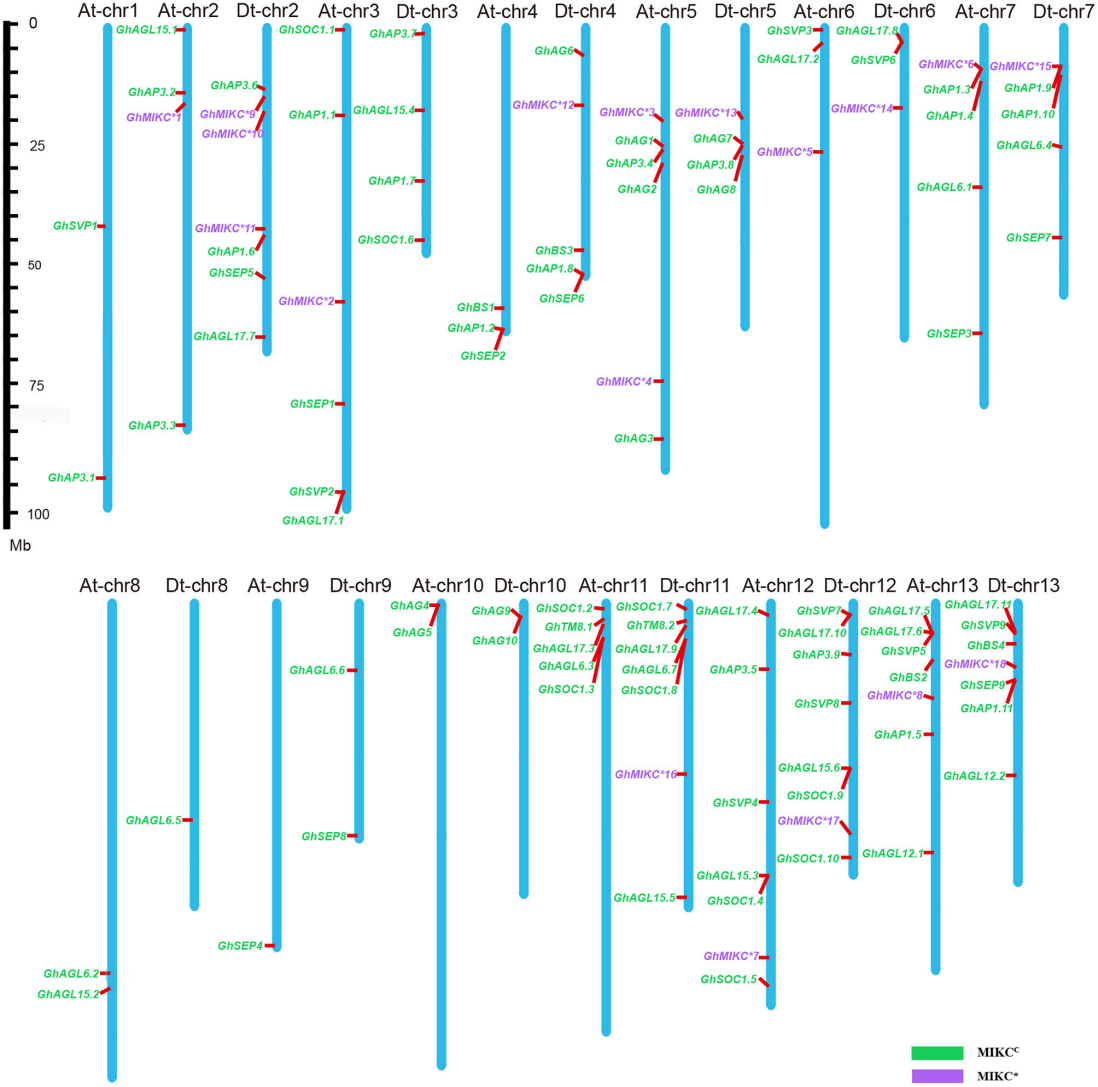
**Chromosomal distribution of *Gossypium hirsutum* L. MIKC genes**. The scale represents megabases (Mbs). The chromosome numbers are shown above each vertical blue bar. Two genes (*GhAP3.10* and *GhAGL17.12* were found on unassembled scaffolds 7,246 and 4,768, respectively) could not be anchored on a specific chromosome. MIKC^*C*^ and MIKC^*^ genes are shown in different colors.

Among the 25 *G. hirsutum* chromosomes, MIKC genes were physically located on all of the 13 A chromosomes and on 12 of the 13 D chromosomes (Figure 4). Among the 110 MIKC genes, two genes, *GhAP3.10* and *GhAGL17.12*, could not be distributed on the *G. hirsutum* chromosomes, but were located on unmapped scaffolds (7,246 and 4,768, respectively). The greatest numbers of genes were located on Dt-chr12 (eight genes), followed by Dt-chr2, At-chr12, At-chr13, Dt-chr11, and Dt-chr13 (seven genes on each). In contrast, two genes were located on chromosomes At-chr1, At-chr8, At-chr10, Dt-chr9, and Dt-chr10. Only one gene was mapped on At-chr9 and Dt-chr8, and no genes were located on Dt-chr1.

### Expression pattern analyses of MIKC genes

**Figure 5:**
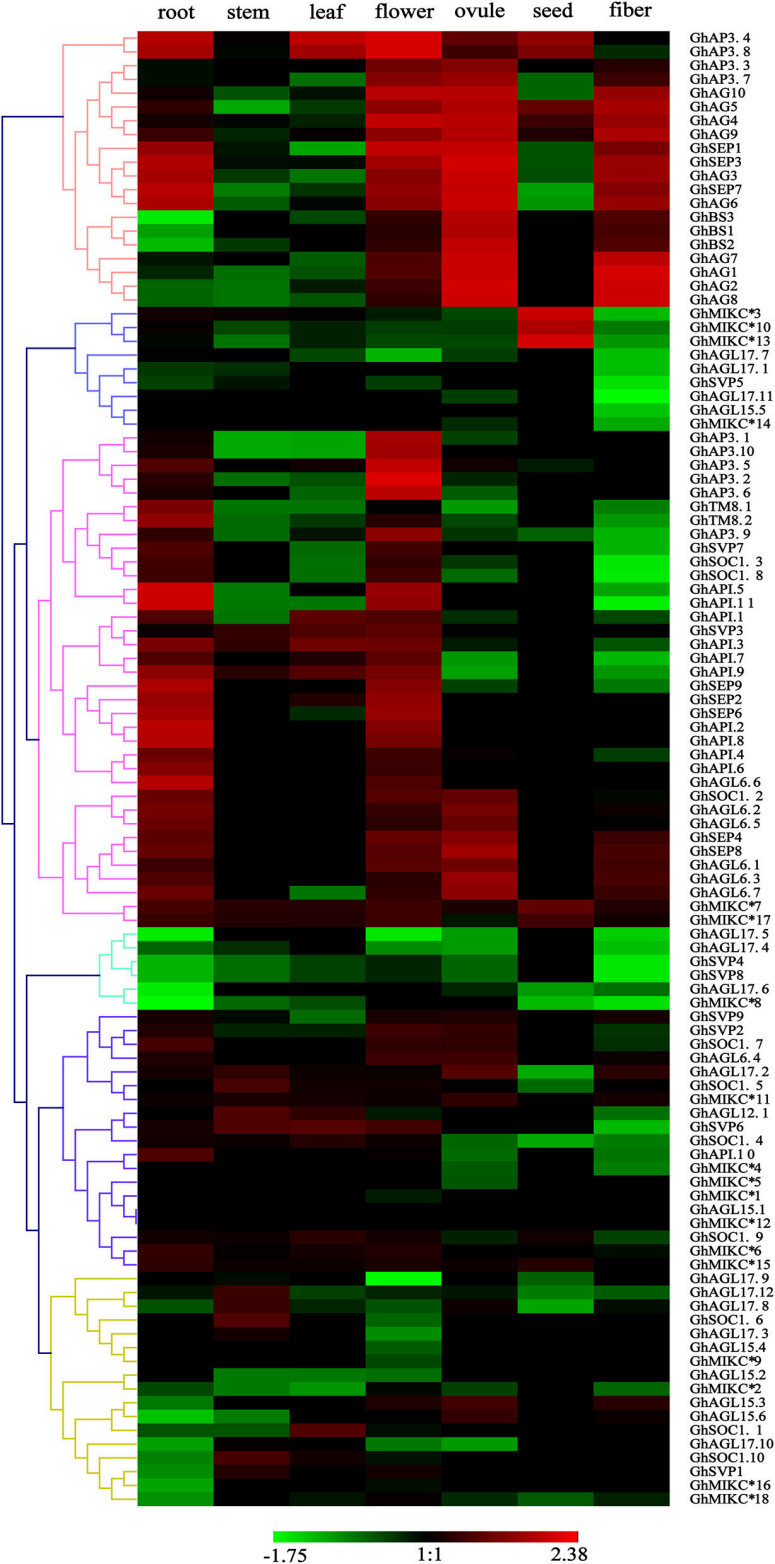
**Heat map showing the hierarchical clustering of expression levels of *Gossypium hirsutum* L. MIKC genes in seven different tissues**. The relative gene expression data was normalized. Gene names are displayed to the right of each row. Cluster analyses of gene expression levels with different color scales are displayed at the bottom.

To explore the expression patterns of the MIKC family genes in *G. hirsutum*- specific developmental processes, the 110 genes' expression profiles were detected in seven different tissues (root, stem, leaf, flower, ovule, seed, and fiber) by transcriptome sequencing (Figure 5). A heat map showed that different genes shared similar expression patterns within subfamilies. For example, the *SEP, AG, AP1, AP3/PI, TM8*, and *AGL6* subgroups were preferentially expressed in flowers. Similarly, the *SVP* subfamily was expressed especially in flowers. Additionally, some *SEP* members (*GhSEP1, GhSEP3, GhSEP4, GhSEP7*, and *GhSEP8*), and the *AG* and *BS* subgroups, were highly expressed in reproductive organs (ovules and fibers). Simultaneously, *SEP, AP1* and five of the *AGL6* genes (*GhAGL6.1, GhAGL6.2, GhAGL6.5, GhAGL6.6*, and *GhAGL6.7*) were also detected in roots. Interestingly, the *SOC* family displayed diverse expression profiles. *GhSOC1.3* and *GhSOC1.7* had high expression levels in roots. In addition, *GhSOC1.8* was mainly expressed in flower, while *GhSOC1.1, GhSOC1.4* and *GhSOC1.9* were highly expressed in leaves. *GhSOC1.6* and *GhSOC1.10* were exclusively and highly expressed in stems. The *GhAGL15* (*GhAGL15.2, GhAGL15.4*, and *GhAGL15.5*) and *GhAGL17* (*GhAGL17.7, GhAGL17.9*, and *GhAGL17.11*) subfamilies were relatively highly expressed in roots, and *GhAGL17.2, GhAGL17.8*, and *GhAGL17.12* were expressed in flowers. Four of the GhMIKC^*^ genes (GhMIKC^*^6, GhMIKC^*^7, GhMIKC^*^17, and GhMIKC^*^18) had high expression levels in flowers, and GhMIKC^*^7 and GhMIKC^*^17 were also highly expressed in seeds.

**Figure 6:**
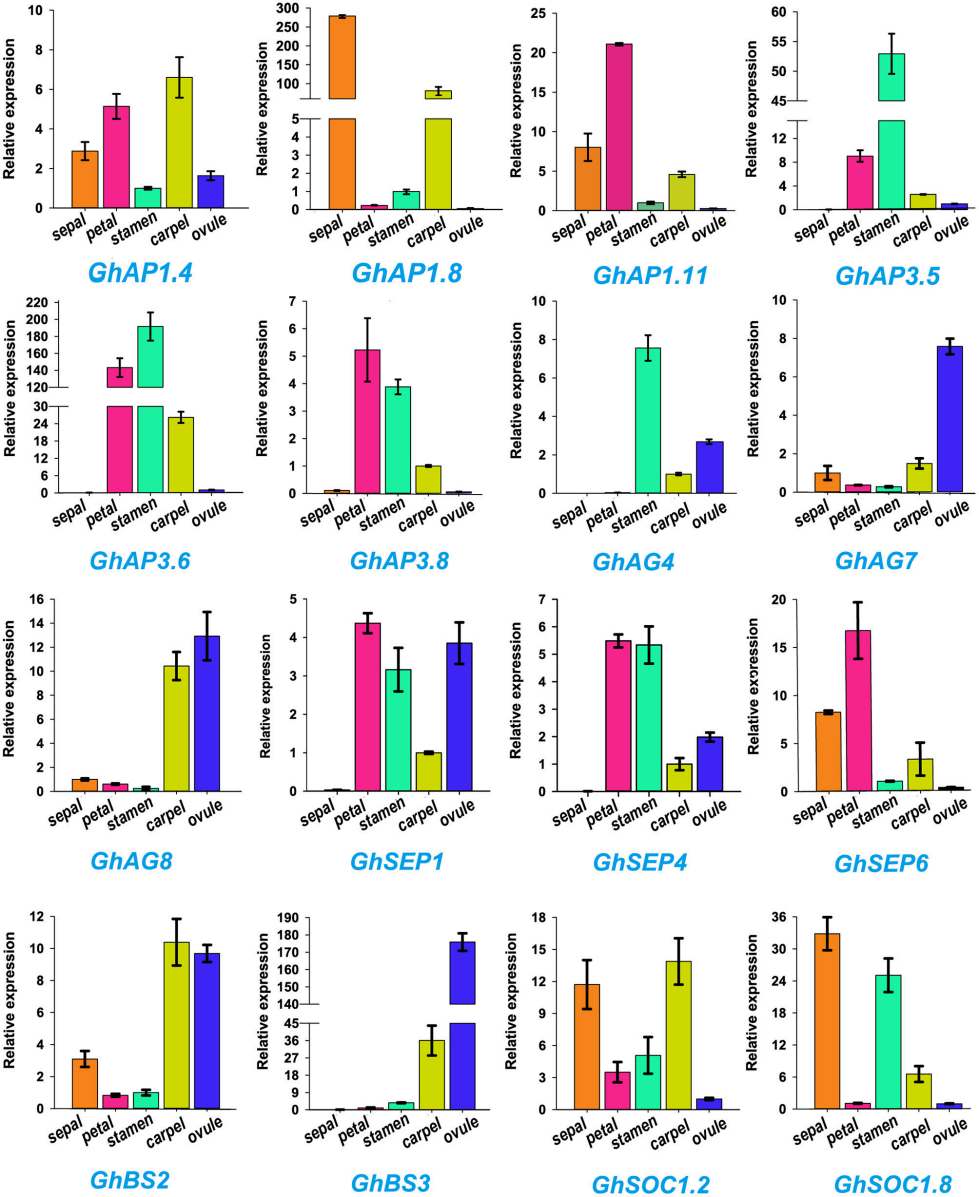
**Expression profiles of 16 *Gossypium hirsutum* L. MIKC genes in five different tissues (sepal, petal, stamen, carpel, and ovule) as determined by qRT-PCR**. The relative expression levels are shown against the reference gene *His3*. Error bars represent the standard deviations of three independent experiments.

ABCDE model genes regulate the formation of five floral organs in Arabidopsis (Sánchez-Fernández et al., 2001; Dietrich et al., 2009; Kuromori, 2010). To validate the participation of MIKC genes in regulating flowering, we selected 16 of ABCDE model orthologous genes to test their expression in five parts of floral organs (sepal, petal, stamen, carpel, and ovule) by qRT-PCR *in G. hirsutum* (Figure 6). *GhAP1.4* and *GhAP1.11 (A class)* showed high expression levels in sepals, petals, and carpel. Differently, *GhAP1.8* was preferentially expressed in sepal. *GhAP3.5, GhAP3.6*, and *GhAP3.8* of the *AP3* subfamily, belonging to *B class*, were expressed in petals and stamens. *GhAG4* of the *C class* displayed the highest expression level in stamen. *GhAG7* and *GhAG8* of the *D class* had higher expression levels in carpel and ovules. *GhSEP1, GhSEP4*, and *GhSEP6 (E class)* were expressed in four different floral organs. *GhBS2* and *GhBS3* (*B sister class*) were mainly expressed in carpel and ovules. *SOC1* accelerates the flowering time, and thus, it is involved in the promotion of floral organ formation. Therefore, high expression levels of *GhSOC1.2* and *GhSOC1.8* were detected in sepals, stamens and carpel. These results were consistent with the ABCDE model.

### Overexpression of the *GhAGL17.9* gene in Arabidopsis

**Figure 7:**
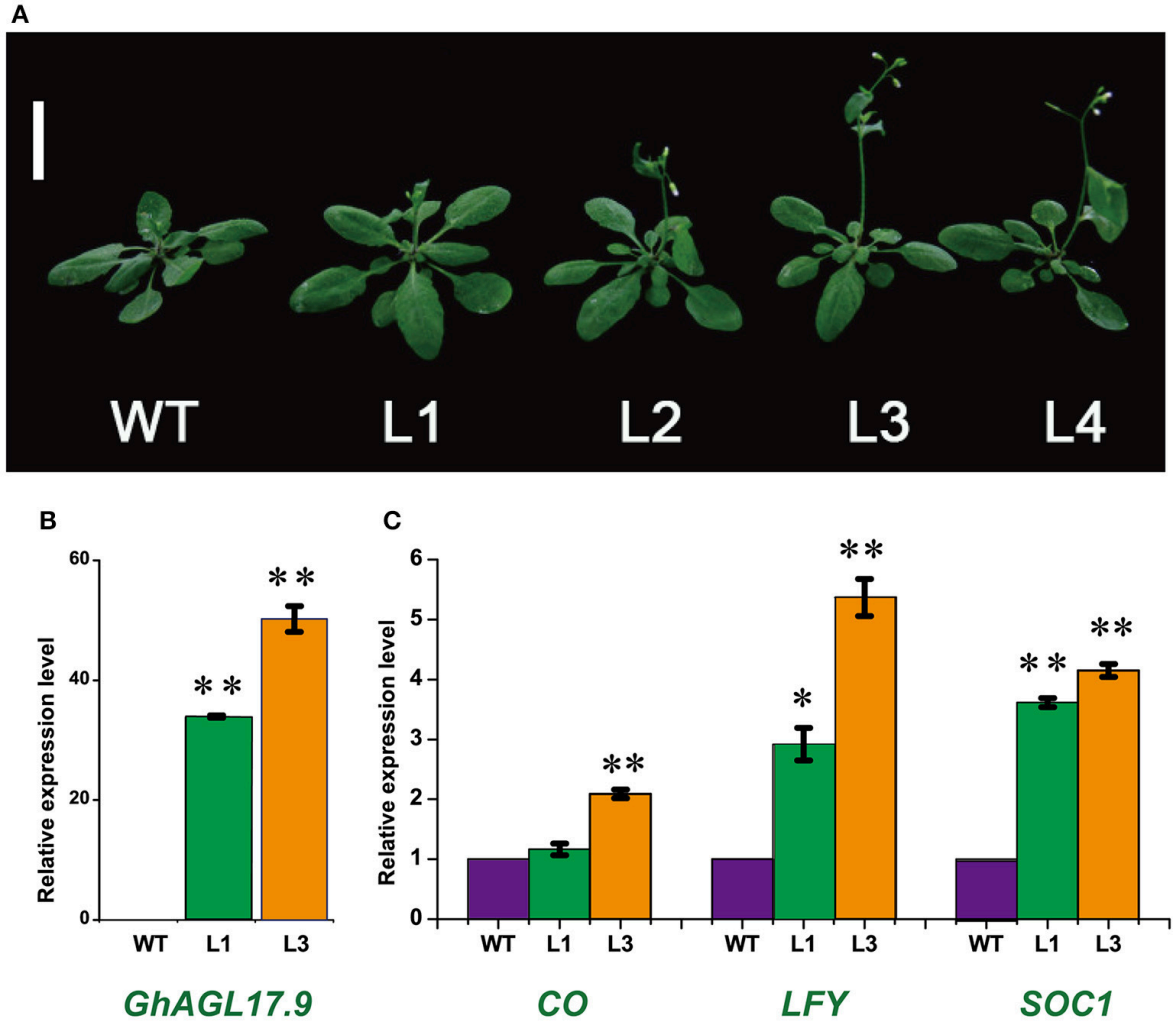
**Phenotypes of transgenic Arabidopsis plants overexpressing *GhAGL17.9* under the *Cauliflower mosaic virus* (CaMV) 35S promoter. (A)**. Morphology of wild type (WT) and transgenic seedlings after 22 days of growth. Bar = 2 cm. **(B)**. A qRT-PCR analysis of *GhAGL17.9 o*verexpression in WT and transgenic Arabidopsis. Significant differences compared with WT (*t*-test):^**^, *P* < 0.01. **(C)**. Expression levels of *SOC1, CO*, and *LFY* as determined by qRT-PCR in WT and *GhAGL17.9*-overexpression plants. *Actin2* was used as the internal control. Error bars represent the standard deviations of three independent experiments. Significant differences compared with WT (*t*-test):^*^, *P* < 0.05;^**^, *P* < 0.01.

AGL17 is the biggest subgroup (Figure 1B). To further investigate the role of the *GhAGL17* subfamily in plant growth and development, we transformed *GhAGL17.9* into Arabidopsis (Columbia-0) driven by the *CAULIFLOWER MOSAIC VIRUS* (CaMV) 35S promoter. We identified 12 T_3_ generation transgentic lines that showed an early flowering phenotype. QRT-PCR results confirmed that *GhAGL17.9* was overexpressed in transgenic lines L1 and L3 (Figure 7). Meanwhile, the numbers of rosette leaves were significantly decreased compared with WT (Table 2). To explore the molecular mechanisms that impact the flowering time in transgentic lines, qRT-PCR was used to detect the expression of flowering-related genes in transgentic lines. LFY is a flowering integration promoting factor, AGL17 can positively regulate the expression of *LFY* gene (Han et al., 2008), and CO is a photoperiod pathway regulator, AGL17 acts downstream of CO (Han et al., 2008). As shown in Figure 7, the expression levels of *LFY* gene in lines 35S-L1 and 35S-L3 were three times higher than in the wild type. *CO* gene expression was not significantly increased. SOC1 is a flowering promoter that regulates different signals of the flowering pathways (Lee and Lee, 2010; Ding et al., 2013). The up-regulation of *SOC1* activates downstream targets, including *LFY* and promotes flowering in Arabidopsis (Schönrock et al., 2006; Lee et al., 2008). Approximate four-fold increases in *SOC1* expression levels were observed in two transgenic lines.

**Table 2:** **Flowering time and the leaf numbers of rosette in WT and p35S::*GhAGL17.9* plants**.

**Genotype**	**Days to the first open flower**	**Rosette leaf number**	***n***
WT	25.37 ± 0.61	8.2 ± 0.94	20
L1	24.29 ± 0.55^**^	7.33 ± 0.9^*^	20
L2	23.72 ± 0.46^**^	5.82 ± 0.73^**^	18
L3	23.83 ± 0.72^**^	5.88 ± 1.17^**^	21
L4	23.81 ± 0.782^**^	5.65 ± 0.7^**^	16

* Represents a significant difference from wild type (t-test, p < 0.05);

** Represents a significant difference from wild type (t-test, p < 0.01);

Data are presented as the mean ± SD;

*Plants were grown under long-day conditions (16 h of light/8 h of dark)*.

## Discussion

Cotton, as an oil crop, plays an important role in agriculture and industry all around the world (Houhoula et al., 2003; Waheed et al., 2010; Mujeli et al., 2016). Floral organs developments affect the yield and quality of cotton seed. The MIKC family members are plant-specific transcription factors containing MADS and K-box domains, and play crucial roles in plant seed development and floral identity (Nesi et al., 2002; De Folter et al., 2006; Mondragon-Palomino and Theissen, 2011). Many MIKC homologs have been analyzed in many plants, including Arabidopsis, *O. sativa, P. tremula, Z. mays, S. bicolor, B. rapa*, and *R. sativus* (Parenicová et al., 2003; Leseberg et al., 2006; Arora et al., 2007; Zhao et al., 2010; Duan et al., 2015; Li et al., 2016). However, the characterization and functional analysis of the MIKC family has not been performed in *G. hirsutum*, an allotetraploid species. In this study, we performed a comprehensive analysis of *GhMIKCs*, which included investigating chromosomal locations, phylogenetic relationships, gene structures, conserved motifs, and expression profiles in different tissues.

### Overall summary of the MIKC family in *Gossypium hirsutum* L

In total, 110 MIKC genes were identified based on *G. hirsutum* genome sequences. Based on phylogenetic relationships with Arabidopsis and *O. sativa* orthologs (Figure S1), the *G. hirsutum* type II MADS family (MIKC^C^) was divided into 13 subfamilies (Figure 1C). Interestingly, an FLC subfamily was not identified in the *G. hirsutum* genome. Similar results were found in *O. sativa, C. sativus, Z. mays*, and *S. bicolor* genomes as well (Arora et al., 2007; Zhao et al., 2010; Hu and Liu, 2012). The *FLC* genes are involved in controlling flowering time through the vernalization and autonomous pathways (Helliwell et al., 2006, 2011; Greb et al., 2007). Vernalization is not required for flowering in *O. sativa, C. sativus, Z. mays*, and *S. bicolor* (Arora et al., 2007; Zhao et al., 2010; Hu and Liu, 2012). Thus, vernalization might not be essential for cotton flowering as well. In addition, we found that in most subgroups, the numbers of proteins in *G. hirsutum* were not doubled, compared with in the diploids Arabidopsis and *O. sativa*. This implied that gene duplication could give rise to the amplification of MIKC subfamily genes in a variety of forms (Flagel et al., 2008; Hargreaves et al., 2014). As previously reported, multiple duplications and diversifications in the different clades of different species cause different evolutionary constraints (Lynch and Conery, 2000; Flagel and Wendel, 2009; Airoldi and Davies, 2012).

Chromosomal assignments indicated that the gene locations were equally divided among four pairs of chromosomes (At-chr6 and Dt-chr6, At-chr7 and Dt-chr7, At-chr10 and Dt-chr10, and At-chr13 and Dt-chr13) in A as well as in D genome (Figure 4). However, five D-genome chromosomes (Dt-chr2, Dt-chr4, Dt-chr9, Dt-chr11, and Dt-chr12) contained more genes compared with the corresponding A-genome chromosomes (At-chr2, At-chr4, At-chr9, At-chr11, and At-chr12). Additionally, large numbers of MIKC genes were located on the last three chromosomes (chr11, chr12, and chr13) of both genomes. This could indicate that the current phenomena were derived from differential rates of genomic evolution and inter-genomic hereditary information transfer (Paterson et al., 2000; Wendel and Cronn, 2003).

### Expression profiles of MIKC genes in *Gossypium hirsutum* L

Global expression patterns analyses in seven different tissues showed that the *API, AP3, AG, SEP*, and *BS* subfamilies were almost all expressed in the flower development stage (Figure 6). Floral organ identities and flower meristem are regulated by five kinds of genetic functional genes (A-B-C-D-E) during flower development, from sepals to ovules (Díaz-Riquelme et al., 2009; Na et al., 2014). A qRT-PCR analysis showed the expression patterns of the orthologous genes of the ABCDE model in flower organogenesis (Figure 6), which were consistent with previous findings in Arabidopsis (Ó'Maoiléidigh et al., 2014; Xie et al., 2015). Further, the *API* subgroup of *A class* genes were not only expressed in sepals and petals, but also exhibited carpel expression profiles. Before and after pollination, the *API*-like gene may aid in the carpel development in Orchidaceae, which triggered ovary development (Mondragon-Palomino and Theissen, 2011; Acri-Nunes-Miranda and Mondragón-Palomino, 2014). Thus, *AP1* subgroup genes may have similar expression patterns in Orchidaceae and allotetraploid cotton. A few GhMIKC^*^ genes were highly expressed in flowers and seeds, which was in accordance with previous results in Arabidopsis (Verelst et al., 2007) and *O. sativa* (Liu et al., 2013). These results indicated that the expression profiles of MIKC^*^ genes were involved in functional redundancy and conservation in the process of *G. hirsutum* evolution.

### Role of the *GhAGL17* gene in flowering

In Arabidopsis, AGL17 acts as a novel flowering promoter, which is involved in the photoperiod pathway. Under long-day conditions, the overexpression of *AtAGL17* causes early flowering (Han et al., 2008). As the largest subgroup of the *GhMIKC*^*C*^ family, one member of the *AGL17s, GhAGL17.9*, was overexpressed in Arabidopsis to explore its biological functions. The transgenic lines displayed earlier flowering than wild type (Figure 7). The expression levels of the related positive marker genes, especially *LFY* and *SOC1*, which are involved in regulating the flowering process, were higher in p35S::*GhAGL17.9* lines than in wild type. *LFY* overexpression can prematurely cause plant development and accelerate blossoming processes (Nilsson et al., 1998; Dornelas and Amaral, 2004). AGL17 targets *LFY* to promote flowering (Han et al., 2008). *SOC1* encodes a MIKC protein, a floral pathway integrator, which is regulated by a variety of flower signaling pathways (Lee et al., 2000; Wang et al., 2009; Ding et al., 2013). However, the relationship between AGL17 and SOC1 in flowering is not clear, which remains to be functionally explored further in the future.

## Conclusions

In this study, 110 MIKC genes were first identified in the *G. hirsutum* genome. The family was divided into 13 subgroups based on a phylogenetic tree, exon/intron structures, and the distributions of conserved motifs. Chromosomal locations of MIKC gene family members were also determined. Finally, the expression patterns of *GhMIKC*s were explored using transcriptome sequencing and qRT-PCR, which revealed the expression levels at different developmental stages. Most MIKC^C^ genes were highly expressed in the floral organs, which was consistent with the ABCDE model. The overexpression of *GhAGL17.9* in Arabidopsis resulted in early flowering through the upregulated expression of *SOC1, CO*, and *LFY*, which suggested that *GhMIKC*s play vital roles in cotton flowering. Our work provides functional insights into the roles of *GhMIKC* genes in cotton flowering.

## Author contributions

ZuY and FL conceived and designed the experiments. ZR and DY performed the experiments. ZhY conducted the phylogeny analysis. CL and LL prepared the materials. HZ and QC analyzed the data. ZR and ZuY wrote the paper. GQ, YL, JL, ZL, and LW helped to revise the paper. All authors read and approved the final manuscript.

### Conflict of interest statement

The authors declare that the research was conducted in the absence of any commercial or financial relationships that could be construed as a potential conflict of interest.
