# Supplementary material for: Genome-Wide Identification of the MIKC-Type MADS-Box Gene Family in Gossypium hirsutum L. Unravels Their Roles in Flowering
Source: Front Plant Sci. 2017 Mar 22;8:384. doi: 10.3389/fpls.2017.00384 (PMC5360754; doi:10.3389/fpls.2017.00384)
Supplement: Supplementary file 1 [file Table1.docx]

**Table S1.** MIKC genes distribution in *Gossypium hirsutum* L.*,* Arabidopsis, *Oryza. sativa* L. and *Vitis vinifera* L.

| Category | Arabidopsis | Rice | Grape | *G. hirsutum* |
| --- | --- | --- | --- | --- |
| MIKC^C^ | 39 | 37 | 37 | 92 |
| MIKC* | 7 | 4 | 7 | 18 |
| Total MIKC genes | 46 | 41 | 44 | 110 |
