# Supplementary material for: Genome-Wide Identification of the MIKC-Type MADS-Box Gene Family in Gossypium hirsutum L. Unravels Their Roles in Flowering
Source: Front Plant Sci. 2017 Mar 22;8:384. doi: 10.3389/fpls.2017.00384 (PMC5360754; doi:10.3389/fpls.2017.00384)
Supplement: Supplementary file 2 [file Table2.DOCX]

**Table S2.** Sequences of the primer pairs used in the qRT-PCR analysis of *GhMIKC* genes and internal reference *GhHis3* gene

| **Gene name** | **Primers for qRT-PCR (5′–3′)** |
| --- | --- |
| *GhHis3* | F: TCAAGACTGATTTGCGTTTCCA |
|  | R: GCGCAAAGGTTGGTGTCTTC |
| *GhAP1.4* | F:GGACCTGCAACTTTTGGAACAACA |
|  | R:TGTCGGTGTCTGCTGTTTTTCTTT |
| *GhAP1.8* | F: TCAACATCTTTCCTGCTGCCT |
|  | R:TCCTCACTTGATCAGTTGCTTCT |
| *GhAPI.11* | F: GGGAAGCTCTTTGAATACTCCACTG |
|  | R: GGACCAGTTGCCCTGAGATTC |
| *GhAP3.5* | F: ATCAGGCAGAGGATGGGTGG |
|  | R: TTGTGTGTGTCGGTTTGCGTT |
| *GhAP3.6* | F: TGCTAGTTCTGGGAAGATGCATGA |
|  | R: CTCCCCTTTCAAATGCCTGAGC |
| *GhAP3.8* | F: CCCTTCCACCACAACGAAGC |
|  | R: GCGCAGGTTCCTGTTAACCTC |
| *GhAG4* | F: CGTGGTCGACTCTATGAGTATGC |
|  | R: CCCCTATGGACTCTCCCAGC |
| *GhAG7* | F: TGTCTCCGAACCAAGATTGCAG |
|  | R: TGGGAGTAAGCAGATGGATGCT |
| *GhAG8* | F: GAGGTACAAGAAGGCATGTTCCG |
|  | R: TGAATCTGCTGCCTCAACTTGG |
| *GhSEP1* | F: ACTAGGGAGGCTCTGGAGCTA |
|  | R: AGCTGCCTCTCAAGTGACTCAA |
| *GhSEP4* | F: TGCTGAGGTTGCCCTTATCG |
|  | R: TCACTGATTGATCGGCTGTTGT |
| *GhSEP6* | F: ACTTTCCATCCTTTGTGATGCTG |
|  | R: CCTGGTAGTTTCTCTGTGTCTCA |
| *GhBS2* | F: GGGACTCGCATTCCTGAGCAT |
|  | R: GTTGATCGAGCTCCTCGTAGGG |
| *GhBS3* | F: ACCCATGAGTTGTCTGTGCTT |
|  | R: TGTTCCCGATTGTCATGCTCA |
| *GhSOC1.2* | F: GATGTCCAGAGTAACATCCCTGAAT |
|  | R: AGCTGCGTTCAAGCTGGTTTTC |
| *GhSOC1.8* | F: TTTGTGATGTTGAGGTTGCTCTT |
|  | R: CTTCAGATGCTGCATGCTTTGT |
