# Supplementary material for: Genome-Wide Identification of the MIKC-Type MADS-Box Gene Family in Gossypium hirsutum L. Unravels Their Roles in Flowering
Source: Front Plant Sci. 2017 Mar 22;8:384. doi: 10.3389/fpls.2017.00384 (PMC5360754; doi:10.3389/fpls.2017.00384)
Supplement: Supplementary file 3 [file Table3.DOCX]

**Table S3.** Sequences of the primer pairs for the flowering marker genes *SOC1*, *CO* and *LFY* and internal reference *Actin2* gene

| **Gene name** | **Primers for qRT-PCR (5′–3′)** | |  |
| --- | --- | --- | --- |
| *Actin2* | | F: GTGTTATGGTTGGGATGGGTCAG | |
|  | | R: CAGGAGCAACACGGAGTTCATT | |
| *CO* | | F: GGAGAGAACAACAGGGCACGA | |
|  | | R: CGGACACGTTTATGGCGGGA | |
| *LFY* | | F: TTTGGGATGCGACTTGGTGG | |
|  | | R: GCTCCTCGTCCTTCATACCCA | |
| *SOC1* | | F: TTGAGCTCTCAGTGCTTTGTG | |
|  | | R: CATGTTTGCTGCTTCATATTTC | |
