# Supplementary figures and images for: Genome-Wide Identification of the MIKC-Type MADS-Box Gene Family in Gossypium hirsutum L. Unravels Their Roles in Flowering
Source: Front Plant Sci. 2017 Mar 22;8:384. doi: 10.3389/fpls.2017.00384 (PMC5360754; doi:10.3389/fpls.2017.00384)

Fig. S1. Phylogenetic relationship between *Gossypium hirsutum* L. and Arabidopsis

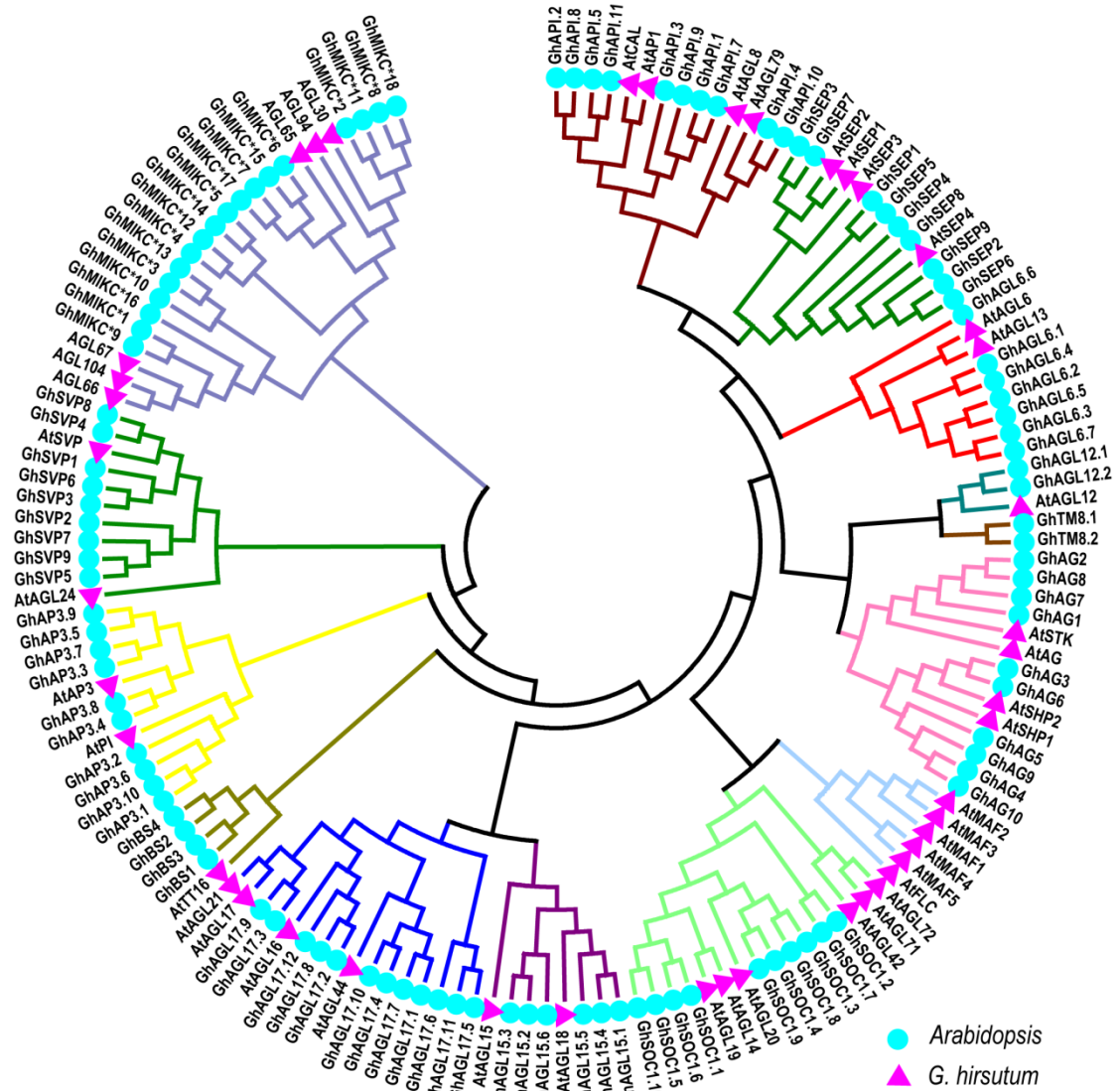

Supplement: Supplementary file 5 [file Image1.PDF]
