# Supplementary material for: Genome-Wide Identification of the MIKC-Type MADS-Box Gene Family in Gossypium hirsutum L. Unravels Their Roles in Flowering
Source: Front Plant Sci. 2017 Mar 22;8:384. doi: 10.3389/fpls.2017.00384 (PMC5360754; doi:10.3389/fpls.2017.00384)

Fig. S2. Sequence logos of MADS domain in *Gossypium hirsutum* L.. The overall height of each stack indicates the sequence conservation. The height of letters represents the relative frequency of the each amino acid at that position

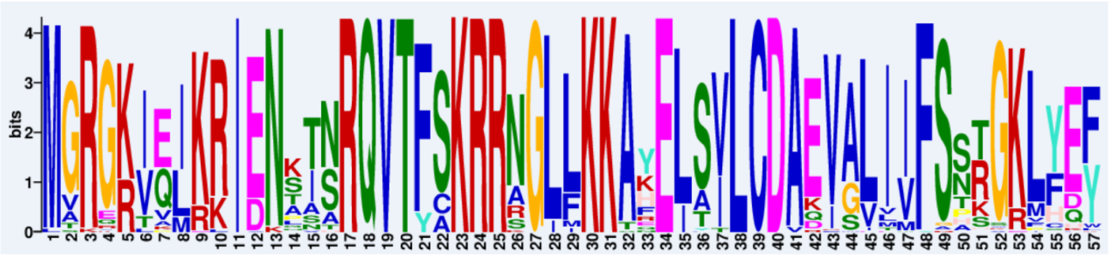

Supplement: Supplementary file 6 [file Image2.PDF]
