# Supplementary material for: Genome-Wide Identification of the MIKC-Type MADS-Box Gene Family in Gossypium hirsutum L. Unravels Their Roles in Flowering
Source: Front Plant Sci. 2017 Mar 22;8:384. doi: 10.3389/fpls.2017.00384 (PMC5360754; doi:10.3389/fpls.2017.00384)
Supplement: Supplementary file 7 [file Image3.PDF]

Fig. S3. The protein sequences of 110 *GhMIKC* genes

>GhSVP1
MAREKIKIKKIDNLTARQVTFSKRRRGLFKKAEELSVLCDAEFALIIFSATGKLFEFATSSMKDILGRYNLHSNKLDQ
PTLELRLENTNEISLSKEVADKTHQMRQMRGEDLQGLNIDELQQLEKLLESGLTRVLETKVHIC
>GhAP3.1
MGRGKIEIKRIENSSNRQVTYSKRRNGIMKKAKEITILCDAKVSLIIFGSSGKMHEYCSPSTNLVDILDQYQKTSGK
KLWDAKHEAISFKAQNNNGCNRSQCKIYLVHDGSSRNLSNEIDRIKKENDNMQIELRHLKGEDITSLPYKELMAI
EDALENGLTYVRGKQDIKAYQSLFSFSFVQMDVLDKTWKNTKFLEEDYKQLSFILNQQQMAYESAREQMDHGY
QRARDYNSQMSSTFQVQPMQPNLQERM
>GhAP3.2
MGRGKIEIKRIENSSNRQVTYSKRRNGIMKKAKEITVLCDAKVSLIIFASSGKMHEYCSPSTKLIDILDQYQKTSGK
KLWDAKHENLSNEIDRIKKENDSMQIELRHLKGEDITSLPYKELMALEDALENGLTCVRAKQMDVLDMAKKNT
KFLEEDNKQLNFIVNQQQLTYENVREHMDHHGYHQAARADFNSQMPFAFRVQPMQPNLQERM
>GhAP3.3
MGRGKIEIKKIENATNRQVTYSKRRNGLFKKAQELTVLCDAKRMEENYRRLKEINKKLRREIRQRMGGDLNELNI
KELQALEAKMDSSLLAIRERKYHVIKTQTDKHKKKVRNLEERHANLVMDLEAKLDGQDGIVETGGYYESTMGLL
PNAASNLYALRLYQNQQPPLVLHDGTNDLRLA
>GhAGL15.1
MGRGKIEIKKIENLNSRQVTFSKRRNGLLKKARELSILCDAEVSVIIFSTTGKLYQWSSTSMEDTLLKYNRGKIVEQ
HPFDEQKAEQNSQSVDVSTLKEEYLKLRAAYMRLNGKELEGLSFKEVQQLEHQLNEGILSIKEHKVFTNQISFS
>GhAPI.1
MGRGRVQLKRIENKINRQVTFSKRRSGLLKKAHEISVLCDAQVALMVFSSKGKLFEYATESCMERILERYERYSFT
EIQCATDEIQQNGNWTWEHAKLKARMETLQRNLRHYEGEDIQNLSLRELQNLEQQLDSALKRIRSKKNQLMV
ESISELQKKDKELQEQNNILAKKLKEKEKTNVEQAHWQLNNNCQDSSSMLLPLNISSNGREKEDNETTNSGVLL
PWMIRHHLE
>GhSEP1
MGRGRVELKRIENKINRQVTFAKRRNGLLKKAYELSVLCDAEVALIIFSNRGKLYEFCSSSSMMKTLERYQKCSHG
APETNVSTREALELSSQQEYLKLKARYEALQRSQRNLLGEDLGPLSSKELESLERQLDSSLKLIRSTRTQYMLDQLT
DLQRKEHLLNEANKSLKQRLMEGYQVHSLQLNPNADDVGYGRQPTHQPQGDVFFHPLDCEPTLQIGYQPDTI
SAVTGCPSVNNYMTGWLP
>GhSVP2
MTRKRIQIKKIDNVAARQVTFSKRRRGLFKKAHELSVLCDAEIALIVFSTTGKLFDYSSASMEKVIERRNQQSGKG
IDRAVTSPYHGLQVGSRTCVMLSKEMAEKTHQLRQLKGEELQGLGYEGLNHLEKLVEGGLRRVTETKDERFFKE
ISTLKMKGAELVEENQQLKQQMENLPHMVHVQPSESIAHAGSSENPIQPYDNSQDISLTLG
>GhAGL17.1
MGRGKLVIRRIDNSTSRQVTFSKRRNGLLKKARELSILCDAEVGLIIFSSTGKLYDYASTSMRSVIERYNRTKEEHH
HQMNPASEVKFWQREVASLRQQLQYLQEYHRQLMGEELSGLSINDLQNLENQLEMSLKGVRMKKDQILTDE
VKELNNKGHLIHQENLELHKKLDLMCQENTELQKKVYGTRQANEASRSSPPNYTFNNGYDLHAPVHLQLSQPL
PQKNDAPEKPMKLG
>GhSOC1.1
MVRGKTQMKRIENAASRQVTFSKRRNGLLKKAFELSVLCDAEVALIIFSPRGKLYEFSSSSMSKTIERYEKREKDNI
GINNKLAAVDQNTQNVKEDAQSMAKKIELLEISKQKLLGKGLEPCSLNELNQLETNLEQSLSRIRERKNLLFRQQI
EKLKQEEKRLKEENAKLRQTCGMNPSPWSTSTEEETMEVETELFIGPPERRRIQNP

>GhBS1
MGRGKIPIKRIENQTTRQVTFSKRRAGLLKKTHELSVLCDAQIGLIIFSTTGKMCQYCTEGYRMEQIIERYQKVTG
TCIPEHDNREHLYNELAVLRKETRRLQLSMRRYTGEDMSSIPFEELDQLEHELERSVIKVRERKNELLQQQLDNL
RRKRKKTAICTAGCRNTERRSSISREEWKLSQWSISKLWINSLSLENQAVCFSLLPFLNSSNPISSSLLSPTFKILMS
SIVPLQLW
>GhAPI.2
MGRGRVQLKRIENKINRQVTFSKRRAGLLKKAHEISVLCDAEVALIVFSHKGKLFEYSTDSWVLFAIFCRHYMGE
DLDSLSLKELQNLEQQLDTAIKHIRSKKNHLISESISELQRKEKAIQEQNAMLAKQIKDREKTVAQAQQSQWGQ
QQQQLGLNTPTSFLLPQPPHPCLNIGGTYQEEATDQVRRNELDLTLEPIYTCHLGYFAA
>GhSEP2
MGRGKVELKRIENKINRQVTFAKRRNGLLKKAYELSILCDAEVALIIFSNRGKLYEFSSSNSIADTLERYNRCTYGAL
EPGQTEIETQQRNYQDYLKLKAKVEVLQHSQRHFLGEELGDLGSKELEQLEHQLDFSLKKIRSLKMEHMVEQLS
KLERKEEMLLETNRNLRRKLDENASTLRSTWETGEQSVPCNLQRPPFFEPLQCTTSMQISYNVPADLTHENIATT
TSAPSGFIPDWML
>GhAG1
MGRGKIEIKRIENTTNRQVTFCKRRNGLLKKAYELSVLCDAEVALIVFSSRGRLYEYSNNNIRSTIDRYKKACSDTS
NTNTVTEINAQYYQQESAKLRQQIQMLQNSNRHLMGDSLSSLTVKELKQVENRLERGITRIRSKKHEMLLAEIEF
LQKREIELENESVCLRTKIAEIERLQQANMVTGPELNAIQALASRNFFSPNVIEHPSAYSHLSDKKILHLG
>GhAP3.4
MARGKIQIKLIENSTNRQVTYSKRRNGLFKKANELTVLCDARVSIIMFSTTGKLHEFISPSTTTKQVIDQYQKTLGI
DIWNTHYEKMQEQLKQLKEVNRNLRKEIRRRMGDCLNDLSIEDLGALEQEMESSVTLIRDRKYRVLSNQIDTSR
KKVRNVEEIHKNLLHELESLKEDPYGLVDNGGDYDTLIGYQNGGPRIFALRLQPNHPSLHSGGGSDLTTYPLLD
>GhAG2
MGRGKIEIKRIENTTNRQVTFCKRRNGLLKKAYELSVLCDAEVALIVFSTRGRLYEYSNNNIRSTIERYKKACSGTS
NTNTVTEINAQYYQQESAKLRQQIQMLQNSSRHLMGDSLSSLTVKELKQLENRLERGITRIRSKKHEMLLAEIEY
FQKREVELENESVCLRAKIAEIERVEEANMVTGAELNAIQALASRNFFTPNVIERGTPTPYSHHDKKILHLG
>GhAG3
MEFPNLDPESSSQKKMGRGKIEIKRIENTTNRQVTFCKRRNGLLKKAYELSVLCDAEVALIVFSSRGRLYEYANNS
VRATIERYKKACSDATTPGSVAEANIQFYQQEATKLRRQIRDVQNMNRHILGEALSSLTFKELKNLEGRLEKGICRI
RSKKNELLFAEIGFMQKREVELQNDNMYLRAKIAENERAQQQSNQLIQAASSYNRNFLPVNLLEPSNNDYSNQ
DQTPLQLV
>GhAGL17.2
MAFLYANDNQGQNDVSETSVCLLSLTPVPHFLSKGKIVIRRIDNSTSRQVTFSKRRNGLLKKAKELAILCDAEVGV
TIFSSTGKLYDFASTSMRSIIERYNKAKEEHQQLGSPTSEVKFWQREAAILRQKLQNLQENHRQMMGEELSGLG
VKELQNLESQLEMSLRGVRIKKDQILMNEIQELNRKGNIIHQENVELYKKVYGTRDVDGANKDSLLTNGLGIGE
DSQVPVCLQLCQPQQQSYETPTRATNLGLVKLQKNSKYGSITNKILKRVYLLIESYVFSFITL
>GhSVP3
MAREKIKIKKIDNLTARQVTFSKRRRGLFKKAEELSVLCDAEVALIIFSATGKLFEFASSSMKDILGRYNLHSNNINK
LDQPSLDLQLENNNNIRLNKEIVDKTRQLRQMRGEDLQGLNVEELQQLEGMLESGLKCVLETKSNRIMNEISSL
ENKGARLLEENKQLKEKVATLYKRKRDSDVVGEEGVSSESVTNVCSCSSSCPPLEDDSSDTSLRLGLPFT
>GhAPI.3
MGRGRVQLKRIENKINRQVTFSKRRSGLLKKAHEISVLCDAEVALIVFSTKGKLFEYSSDSCMERILERYERYSYSE
RQLAANENERTGSWTLEHAKLKARMEVLQRNQRHYMGEDLENLSLRELQNLEHQLDSALKHIRSRKNQLMFE
SISELQKKDKALQEQNNVLAKKVKEKEKEMAHQPQQNNCQDSSSMLPQPLQSLNTSDTNEARSNGREEGNP

SPAQHRNSNVLLPPWMIPRIE
>GhAPI.4
MGRGRVQLRRIENNISRQVTFSKRRSGLLKKANEISVLCDADVALIVFSNKGKLFEFSSDPSMERILERYERQIYAP
TGSESQANWSLESSKLMSTIEVLQRNLRNFRGEELEPLSSRDLQLLEQQIGNSLKRIRTRKNKLMNESISVLQKRE
KTLQDQNNMLAKKLKEKQQTPTEHAQHEVQQKLVQNSPPSTSIQPPTPPPAATRFPCLTIGGSYEAMKGTNKE
AELNLNLVPNQ
>GhAGL6.1
MGRGRVELKRIENKINRQVTFSKRRNGLLKKAYELSVLCDAEIALIIFSNRGKLYEFSSSGMTKTLERYQRCCFIPH
DNTHERETQSWYQEVIKLNAKYEALQRTQRHLLGEDLGPLNMKELHNLEKQLEGALARARQRKTQIMMEQM
DDLRKKERQLGDLNKQLIIKLEAEGQNLETIQGLWGCCGAAATENFPLHLSQTQPMECDLQPVLQIGYHHYVEA
EGSSAPKDMAGETNFIHGWALQYPFEPSRPSCRLIVALIYKLFNIVLVRTYYSINLS
>GhSEP3
MGRGRVELKRIENKINRQVTFAKRRNGLLKKAYELSVLCDAEVALIIFSNRGKLYEFCSSSSMLKTLDRYQKCSYGA
VEVSKPAKELESSYREYLKLKARYEELQRTQRNLLGEDLGPLNSKELEQLEHQLESSLKHVRSTKTQYMLDQLTEL
QNKEQMLMETNRALSIKLEEISARNQFRASWEGGEQSVAFTNQQAQSMGLFQPLECNPTLQIGYCNPVASD
QMAATTHAPQVNGFIP
GWML
>GhAGL6.2
MGRGRVELKRIENKINRQVTFSKRRNGLLKKAYELSVLCDAEVALIIFSNRGKLYEFGSSGMTKTLERYQQCCFTP
QPQHNIPEHETQSWYQEIIKLKAKYEALERTQRHLLGDDLGPLNLKELQNLEKQLEGTLVLARQRKTQIMMEQ
MEDLRKKERQLGELNKQLKIKLDGEGQNLKTSQGLWSCCTTAENSHIPLHPSHPNPMECDHEPVLQIGYHHHY
VEAEGSSVPRSMAGETNFIHGWVI
>GhAGL15.2
MGRGKIEIKRIENANSRQVTFSKRRAGLLKKAKELAILCDAEVAVIIFSNTGKLFEFSSSGMKKTFSRYNKCLQGSI
EMALVEHKAEKQVCKEADNLKDEVAKLQMKQLQLLGKNLTSVSLKELEVLEQQLSEGLSSVKEKKEQLLMEQLE
RSRLQVCQVPLCMSCYIYRERLEELRGFFPSTDHLVRSYLEYYPVERKNSLMSHNIRGPDVTCACNLEKGDSDTTL
YLGLPSDHHKIKKPESHSNDSESQ
>GhSEP4
MGRGRVELKRIENKINRQVTFAKRRNGLLKKAYELSVLCDAEVALIVFSNRGKLYEFCSSPSMTKTLEKYQKCSYS
TLDNSRSISETQNSYQEYLKLKARVEVLQTSQRNLLGEDLGPLDSKELDQLEHQLEASLKQIRSTKVQAMLDQLN
DLHNREKLLMDANKSLRRKLEELSTQVPQGPAWDNIGGPCIPPYNHLTEAQSEAFFHPLGANCSSQIGYSNDV
VSDEMNAAVHSQNVNGYFPGWML
>GhAG4
MMLLLSSWFFFWFFGLFQLGSMVYPNESLEDSPQKKMGRGKIEIKRIENTTNRQVTFCKRRNGLLKKAYELSVL
CDAEVALIVFSSRGRLYEYANNSVKATIERYKKASDSSNTGSVAEVNAQFYQQEADKLRNQIRNLQNANRHMLG
ESIGGLPMKELKSLESRLEKGISRIRSKKNELLFAEIEYMQKREIDLHNNNQLLRAKIAENERKQQSMNLMPGGS
SANFEALHSQPYDSRNYFQVDALQPATNYYNPQQQQDQIALQLV
>GhAG5
MVYPNESLEDSPQKKMGRGKIEIKRIENTTNRQVTFCKRRNGLLKKAYELSVLCDAEVALIVFSNRGRLYEYANN
SVKATIERYKKASDSSNTGSVAEVNAQFYQQEADKLRNQIRNLQNTNRHMLGESVGGLPMKELKSLETRLEKGI
SRIRSKKNELLFAEIEYMQKKEIDLHNNNQLLRAKIAENERKQESMNLMPGGSSNNFEAIHSQPYDSRNYFQVD
ALQPAANYYNPQQQQDQIVLQLV
>GhSOC1.2
MVRGKIQMKRIENATSRQVTFSKRRNGLLKKAYELYVLCDAEVAVIIFSHKGKLYEFSSSDNMQNTIERYRQYKK

DVQSNIPEFDRYTQQLRLEAENMAKKIEFLEVSKRRMLGQNLGSCSIDELQEVENQLERSLRNIRARKGYLFKEQ
ILQLKAKERYMQEENAKLSAKNNGTTCSQQNAEVETELFLGLPENRCS
>GhTM8.1
MGRGKVQLKRIENPTNRQVTFSKRRNGLLKKAFELSILCDAEVALLIFSSSGKVYQFASHDMDRTVAKYRREVGL
PDSSNPQFRTMEFWRSEIDELNRSINTLEARLKYVQLISQYLNIYIFHVFRTSNVFDFCRHLSGEDILALGMRDLK
QLERQLKIGVERVRSRKRRIVSDHATLLKRRHKQLQEENSRLHKRLKELQDGNISSGLVGENACTMFHQRIVHEE
DFHNETGLPL
>GhAGL17.3
MGRGKIAIRRIDNSASRQVTFSKRRKGLIKKAKELAILCDAEVGLVIFSSSGKLYEFASTSMKTVIERYNTLKEEHQ
QLSNPSSEVKFWQREAAILKQQLQNLQDNHRQLMGEQLYGLRVEDLQNLENQLEMSLKGVRMKKERILTNEI
EELNRRGSLIHQENVELFKKDNKKECSDELKRYVTPVHPQQNPCL
>GhAGL6.3
MGRGRVELKRIENKINRQVTFSKRRNGLLKKAYELSVLCDAEVALIIFSSRGKLYEFGSSGMSKTLERYQRCCFTP
QDNSLERETQNWYQEVTKLKAKYEALQRTQRHLLGEDLGPLNVKELQNLEKQLEGALALARQRKTQIMIEQME
DLRKKERELGDLNKQLKIKLEAEGQNLKTIQGLWSSGAAAETSNFPLHPSHPHPMDCDHEPVLQIGYHHFVQA
EGSSVPKSMAGETNFIHGWVI
>GhSOC1.3
MVRGKTQMKRIENPTSRQVTFSKRRNGLLKKAFELSVLCDVEVALIIFSPRGKPYEFASSSMQETIARYLRHTKD
NRVKPTEQSMQHLKTEAEKMLKKIELLEVSRRKLLGENLGSCTLEELQQIEQQLERSVTRVRARKAKVFKDQIEK
LKEKEEVLAAENAKLCEKYGLLPGKGSKEVNENEEANDESNPSSDVETELFIGLPEGRAKRIVQPNSTD
>GhAGL17.4
MGRGKIVIRRIDNSTSRQVTFSKRRNGLLKKARELSILCDAEVGLIIFSSTGKLYDYASSSMKSVIERYNKVKEEHH
QLLNPDSQVKFWQREAASLRQQLQYLQDYHRQLMGEELSGLSVKDLQHLENQLEGSLKGVRTKKEQILTDEIK
ELNHKGHLIHQENLELYKKVDLIHQQNTELQKKIYGTREANEASRISHPNYTFNNGYDLHAPVRLQLSQPQPPK
NNAPAKSMKLG
>GhAP3.5
MGRGKIEIKRIENATNRQVTYSKRRNGIFKKAQELTVLCDAKVSLIMFSSTGKFHEFISPNISTKAFFDLYQKTTGT
DLWISHYEKMQENYRRLKEINKKLRREIRQRMGGDLDDLNIKELQALEAKMDSSLVAIRDRKYHVIKTQTDTHK
KKVRNLEERHANLVFDLETKLDQQNGIVESEGYYNEAANGASNLHALRLYQIHHPNLVLQHGGRFGSNDLRLA
>GhSVP4
MAREKIQIKKIDNSTARQVTFAKRRRGLFKKAEELAILCDADVALIIFSSTGKLFDYASSSMKEILERHHLRSKNLEK
LEQPCLGLKLVEHSNQSMLTMEIAEKSHQLRQMRGEELHGLNIEELQQLEKSLEIGLSRVMEKKGQRIMREIND
LQRKGMQLMEENERLKQQIINGPRQVAGDSDNIIFGEEGQSSESVTNVCTSNGNPHDYESSVTSLKLG
>GhAGL15.3
MGRGKIEIKRIENANSRQVTFSKRRAGLLKKAKELAILCDAEVAVIIFSNTGKLFEFSSSGMNKTISRYKSAQGSPEI
AQVEHKAEKQDSKEADHLKDEIAKLQMKQLQLLGKNLTSMSLKELQLLEQQLNEGLLSVKEKKEQLLMQQLEQ
SRLQEQRAMLENETLRRQVEELRGFFPTTDHPIQPYLECYPVERKNSLMSHSIPSPDLTCNCTVEKGDSDTTLYL
GLPSDYHKRKKPEIESHSNESESQLGLL
>GhSOC1.4
MVRGKTQMRRIENNTSRQVTFSKRRNGLLKKAFELSVLCDAEVALIIFSPRGKLFEFASSSMQQTIERYRRRTKD
NETNKPIEQNLQHLKTESANMLKTLEDLEISRRKLLGENLGSCTLEELQEIEQQLQKSVSIIRARKTQIFRDQIEQLK
EKEKALAAENEKLCEKLFMSQCGTKSWKRLSEQEDNVPYDESSPSSDVETELFIGLPEGRTRRIVQLN
>GhSOC1.5
MGCLDTCSEADGPQPVGPRERVVWRKLKSEKMVRRRTQLKRIENAASRQVTFSKRRNGLLKKAFELSVLCDAE

VALIIFSPRGKLYEFSSSSTNKTIERYQKRQKDIHGISSKGEDMQDDVKEDAHRLAKKIESLEDSKRKLLGHGLEPC
SIDDLLLLEKQLERSLSRIRARKNQVFTEQIEKLKEEERRLGEENANLREECGMRPRESTSTRQSDDERNMEVETE
LCIGPPERRCILKP
>GhAGL17.5
MGRGKIEIKKIEKSSSRQVTFSKRRNGLLKKAKELAILCDAEVGLIIFSSTSKLHHFASSSMKSVIERYTKYEEEYHH
QLLDPASELKFWKKEVASLRQQQNDLQEYQRQLKGKEKELSGLSFKDLQGLENQLEMSLKRVRMRKDQILTNQ
IDELKRKGHHIDQENLKVHKKLDLICHENTELQKKVNGNGTEEANESSKSLSHSYGFNNGYDYLQAPVVDLRLSL
PQQLPDADTSKSKTR
>GhAGL17.6
MGRGKIEIKKIEKSSSRQVTFSKRRNGLLKKAKELAILCDAEVGLIIFSSTAKLHHFASSSMKSVIERYNKYREEYHH
QLLDPASELKFWEKEVASLRQQLNDLQEYHRQLMGEELSGLSIKDLRNLENQLQMSLKSVRMRKDQILTNQIEE
LSLKGHHIHKENLELQKKLDLICQENTELQRKVDGNGTAEANEGSKSSSQSYGFNNGHDELQAPIVDLRLSQPQ
QLPDADTSYRNLSLF
>GhSVP5
MTRQKIQIKKIDNTAARQVTFSKRRRGLFKKAHELSTLCDAEIALLVFSNAGKLFEYSSTSTRQVIERRNLQSERID
RLDPISTLELQLQSSTCAMLGKEIAEKTKELRQLRGEELQGLDLEELKHLEKLLEGGLNRVTQTKDELFFKEISILKR
KEVELMEENQQLKEKMGNSPHVVQPTVAQQGLGQPSECNGHAWRSYSSDISLRLG
>GhBS2
MGRGKIAIKRIENQTTRQVTFSKRRAGLLKKTHELSVLCDAQIGLIIFSSTGKMCQYCTQPYRMEQIIERYQKVTG
TRIPEHDNREHLYNELAVLRKETRRLQLSMRRYTGEDMSSIPYEELDQLEQELERSVNKVRERKNELLQQQLDNL
RRKERMLEEENNNMYRWIQEHRAAIEYQQHGGLEAKPVEHHQQVLDEFPFYGEPSSVLQLATIPQQFSYQLQ
LAQPNLQDSNV
>GhAPI.5
MGRGRVQLKRIENKINRQVTFSKRRAGLLKKAHEISILCDAEVALIVFSHKGKLFEYSTDSCMEKILERYERYSYAE
RQLVASEPESQGNWSMDYNRLKAKVELLQRNHRHYMGEELESLSLKELQNLEQQLDTALKLIRSKKNQLMYESI
SELQRKEKAIQEQNTMLAKQIKEREKTVAQQQQQQPQWGQQDHGLNTSSFLLPQPPPCLNIGGTYQEEATE
MRRNELDLTLEPIYSCHLGCFAA
>GhAGL12.1
MGRGRVQMKRIENPVHRQVTFCKRRAGLLKKAKELSVLCDAEIGVVIFSAHGKLYELATKGTMQELIERYGKYT
GGPPADEPMVEPMDAKKEIEMLKQEIEILQKGLRYMFGGGCEYMSLNELLVLEKHLEIWINHIRSTKMDIMFQ
EIQMLRNKEGIMKAANKCLQETIEENISYTDFTTMTTNMMHPLTIANNIF
>GhAP3.6
MQISKPEIECLKLLVFHSWLLSLNLILCSIPHHFTTKKHQSKEVEREKSKMGRGKIEIKRIENSSNRQVTYSKRRNGI
MKKAKEITVLCDAKVSLIIFASSGKMHEYCSPSTKLIDILDQYQKTSGKKLWDAKHENLSNEIDRIKKENDSMQIE
LRHLKGEDITSLPYKELMALEDALENGLTCVRAKQASIYTHTHTNTYLHTHTHTHTHTHTYKILQNQQQLTYENV
REHMDHHGYHRAARADFNSQMPFAFRVQPMQPNLQERM
>GhAPI.6
MGRGRVELKRIENKINRQVTFSKRRSGLFKKAHELSVLCDAEVALIVFSHKWKLYEYSTDSCMEKILERYERHYYA
ERHLVATEPESQVDLFSLVLSYVISLISFLRLRHYMGEDLDPLSLKELQNLEQQLDTAVKHIRARKNQLLNESISELQ
RKEKAIKEQNAMLANQIKEREKTVARQSQWGLQDNGLNTSSFVLPHPHPSLNIGLISHVLGIRSRI
>GhSEP5
MGRGRVELKRIENKINRQVTFAKRRNGLLKKAYELSVLCDAEVALIIFSNRGKLYEFCSSSSMMKTLERYQKCSHG
APETNVSTREALELSSQQEYLKLKARYEALQRSQRNLLGEDLGPLSSKELESLERQLDSSLKLIRSTRTQYMLDQLT
DLQRKEHLLNEANKLKQRLMEGYQVHSLQLNPNADDVGYGRQPTHQPQGDVFFHPLDCEPTLQIGYQPDTIS

AVTGPSVNNYMTGWLP
>GhAGL17.7
MGRGKLVIRRIDNSTSRQVTFSKRRNGLLKKARELSILCDAEVGLIIFSSTGKLYDYASTSMRSVIERYNRTKEENH
HQMNPASEVKFWQREVASLRQQLQYLQEYHRQLMGEELSGLSINDLQNLENQLEMSLKGVRMKKDQILTDE
VKELNNKGHLIHQENLQLHKKLDLMYQENTELQKKAYGTRQANEASRSSPPNYTFNNGYDLRAAVHLQLSQPL
PQKNDAPEKPMKLG
>GhAP3.7
MGRGKIEIKKIENATNRQVTYSKRRNGLFKKAQELTVLCDAKVSLIMFSSTGKFHEFLSPNISTKGFFDLYQKTTGI
DLWNSHYERMEENYRSLKEINKKLRREIRQRMGGDLNELNIKELQALEAKMDSSLLAIRERKYHVIKTQTDKHK
KKVRNLEERHANLVMDLEAKLDGQDGIVETGGYYESTMGLLPTGASNLYALRLYQNQQPPLVLHHGTNDLRLA
>GhAGL15.4
MGRGKIEIKKIENLTSRQVTFSKRRNGLLKKARELCILCDAEVGVIIFSTTGKLYQWSSTSMEDTLLRYNRGKVVE
QHPSDDQKAEQNSQSFDVSALKEEYLKLRAAYMRLNGKELEGLSFKEVQQLEHQLNEGILSVKEHKVFTNRISIS
>GhAPI.7
MGRGRVQLKRIENKINRQVTFSKRRSGLLKKAHEISVLCDAQVALMVFSSKGKLFEYATESCMERILERYERNSYT
EIQCATDEIQQNGNWTWEHAKLKARMETLQRNLRHYEGEDIQNLSLRELQNLEQQLDSALKRIRSRKNQLML
ESISELQKKDKALQEQNNILAKKEKEKTNVEQAHWQLNNNCQDSSSMLLPLNISSNGREKEDNETTNSGVLLP
WMIRHHLE
>GhSOC1.6
MVRGKTQMKRIENAASRQVTFSKRRNGLLKKAFELSVLCDAEVALIIFSTRGKLYEFSSASMSKTIERYEKREKDN
TGINNKLAAVDQNTQNVKEDAQSMAKKIELLENSKQKLLGNGLEPCSLNELNQLETNLERSLSRIRERKNLLFRQ
QIEKLKQEEKRLEEENANLRQTCGMKPSPWSTSTEEETMEVETELFIGPPERRRIQNP
>GhAG6
MEFPNLDPESSSQKKMGRGKIEIKRIENTTNRQVTFCKRRNGLLKKAYELSVLCDAEVALIVFSSRGRLYEYANNS
VRATIERYKKACSDATTPGSVAEANIQFYQQEATKLRRQIRDVQNMNRHILGEALSSLTFKELKNLEGRLEKGICRI
RSKKNELLFAEIGFMQKREVELQNDNMYLRAKIAENERAQQQSNQLMQAASSYNRNFLPVNLLEPSNNDYSN
QDQTPLQLV
>GhBS3
MGRGKIPIKRIENQTTRQVTFSKRRAGLLKKTHELSVLCDAQIGLIIFSTTGKMCQYCTEGYRMEQIIERYQKVTG
TCIPEHDNREHLYNELAVLRKETRRLQLSMRRYTGEDMSSIPFEELDQLEHELERSVIKVRERKNELLQQQLDNL
RRKRKKTAICTAGCRNTERRSSISREEWKLSQWSISKLWINSLSLENQAVCFSLLPFLNSSNPISSSLLSPTFKILMS
SIVPLQLW
>GhAPI.8
MGRGRVQLKRIENKINRQVTFSKRRAGLLKKAHEISVLCDAEVALIVFSHKGKLFEYSTDSWVLFAIFCRHYMGE
DLDSLSLKELQNLEQQLDTAIKHIRSKKNQLISESISELQRKEKAIQEQNAMLAKQIKEREKTVAQAQQSQWGQ
HQQQLGLNTSTSFLLPQPPHPCLNIGGTYQEEATDQVRRNELDLTLEPIYTCHLGYFAA
>GhSEP6
MGRGKVELKRIENKINRQVTFAKRRNGLLKKAYELSILCDAEVALIIFSNRGKLYEFSSSNSIADILERYNRCTYGAL
EPGQTEIETQRNYQEYLKLKAKVEVLQHSQRHFLGEDLGDLGSEELEQLERQLDFSLKKIRSLKMEHMVEQLSKL
ERKEEMLLETNRNLRRKLDENASTLRSTWETGEQSVPCNLQHPRFLEPLQCTTSMQISYNVPADLTHENIATTTS
APSGFIPDWML
>GhAG7
MGRGKIEIKRIENTTNRQVTFCKRRNGLLKKAYELSVLCDAEVALIVFSSRGRLYEYSNNNIRSTIDRYKKACSDTS
NTNTVTEINAQYYQQESAKLRQQIQMLQNSNRHLMGDSLSSLTVKELKQVENRLERGITRIRSKKHEMLLAEIEF

LQKREIELENESVCLRTKIAEIERLQQANMVTGPELNAIQALASRNFFSPNVIEHPSAYSHPSDKKILHLGYFFFSLR
TNVSKIKLYIYECVKFYII

>GhAP3.8

MARGKIQIKLIENSTNRQVTYSKRRNGLFKKANELTVLCDARVSIIMFSTTGKLHEFISPSTTTKQVIDQYQKTLGI
DIWNTHYEKMQEQLKQLKEVNRNLRKEIRRRMGDCLNDLSIEDLGALEQEMESSVTLIRDRKYRVLSNQIDTSR
KKVRNVEEIHKNLLHELESLKEDPYGLVDNGGDYDTLIGYQNGGPRIFALRLQPNHPSLHSGGGSDLTTYPLLD

>GhAG8

MGRGKIEIKRIENTTNRQVTFCKRRNGLLKKAYELSVLCDAEVALIVFSTRGRLYEYSNNNIRSTIERYKKACSGTS
NTNTVTEINAQYYQQESAKLRQQIQMLQNSSRHLMGDSLSSLTVKELKQLENRLERGITRIRSKKHEMLLAEIEY
FQKREVELENESVCLRAKIAEIERVEEANMVTGAELNAIQALASRNFFTPNVIERGTPTPYSHHDKKILHLG

>GhAGL17.8

MAFLYANDNQGQNDVSETSVCLLSLTPVPHFLSKGKIVIRRIDNSTSRQVTFSKRRNGLLKKAKELAILCDAEVGV
TIFSSTGKLYDFASTSMRSIIERYNKAKEEHQQLGSPNSEVKFWQREAAILRQKLQNLQENHRQMMGEELSGLG
VKELQNLESQLEMSLRGVRIKKDQILMNEIQELNRKGNIIHQENVELYKKVYETRDVDGANKDSLLTNGLGIGED
SQVPVCLQLCQPQQQSYETPTRATNMGYIPLYMLDFDLMQISVPQFDVHLFSW

>GhSVP6

MAREKIKIKKIDNLTARQVTFSKRRRGLFKKAEELSVLCDAEVALIIFSATGKLFEFASSSMKDILGRYNLHSNNINK
LGRPSLDLQLENNNNIRLSKEIVDKTHQLRQMRGEDLQGLNIEELQQLEGMLESGLKCVLETKSNRIMNEISSLE
TKGARLLEENKQLKEKMATLYKRKRDSDVVGEEGVSSESVTNVCSCSSSCPPLEDDSSDTSLRLGLPFT

>GhAPI.9

MGRGRVQLKRIENKINRQVTFSKRRSGLLKKAHEISVLCDAEVALIVFSTKGKLFEYSSDSCMERILERYERYSYAE
RQLAANENERTGSWTLEHAKLKARMEVLQRNQRHYMGEDLENLSLRELQNLEHQLDSALKHIRSRKNQLMFE
SISELQKKDKALQEQNNVLAKKVKEKEKEKEKEKEKEMTHQPQQNNCQDSSSMLPQPLQSLNISDTYEARSNG
REEGNPSAAQHRNSNVLLPPWMIPRIE

>GhAPI.10

MGRGRVQLRRIENNISRQVTFSKRRSGLLKKAHEISVLCDADVALIVFSNKGKLFEFSSDPSMERILERYERQIYAP
TGSESQANWSLESSKLMSTIEVLQRNLRNFRGEELEPLSLRDLQLLEQQIGNSLKRIRTRKNKLMNESISVLQKRE
KTLQDQNNMLAKKLKEKQQTPTEHAQHEVQQKFVQNSPPSTSVQPPTPPPAAIQFPCLTIGGSYEAMKGTNK
EAELNLNLVPNQ

>GhAGL6.4

MGRGRVELKRIENKINRQVTFSKRRNGLLKKAYELSVLCDAEIALIIFSNRGKLYEFSSSGMTKTLERYQRCCFIPH
DNTHERETQSWYLEVIKLNAKYEALQRTQRHLLGEDLGPLNMKELHNLEKQLEGALARARQQKTQIMMEQM
DDLRKKERQLGDLNKQLIVKSPSFMAFSYQYSSPNNLSLPLLRSHFKFSNRPSKSQD

>GhSEP7

MGRGRVELKRIENKINRQVTFAKRRNGLLKKAYELSVLCDAEVALIIFSNRGKLYEFCSSSSMLKTLDRYQKCSYGA
VEVSKPAKELESSYREYLKLKARYEELQRTQRNFLGEDLGPLNSKELEQLEHQLESSLKHVRSTKAPNSTLFYFNLL
LQTQYMLDQLSELQNKEQMLMETNRALSIKLEEVSARNQFRVSWEGGEQSVAFTNQQAQSMGLFQPLECNP
TLQIGYCNPVASDQMAATTHAQQVNGFIPGWML

>GhAGL6.5

MGRGRVELKRIENKINRQVTFSKRRNGLLKKAYELSVLCDAEVALIIFSNRGKLYEFGSSGMTKTLERYQQCCFTP
QPQHNIPEHETQSWYQEIIKLKAKYEALERTQRHLLGDDLGPLNLKELQNLEKQLEGTLVLARQRKTQIMMEQ
MEDLRKKERQLGELNKQLKIKLDGEGQNQKTSQGLWSCCTTAENSHFPLLPSHPNPMECDHEPVLQIGYHHH
YVEAEGSSVPRSMAGETNFIHGWVI

>GhAGL6.6

MGRGKVVLERIENKINRQVTFSKRRNGLLKKSYELSVLCDAEVALIIFSSRGKLSEFASNISVPTTLEKYWQHRYSS
PVDIPLDETQTLYQEVLRLKAKYESLQRSQRHLLGEELESLTVKELYKIEKQLDRALSQARQKKTQLLLERMEELSK
KERELEVENKQLKSQLELEHCFQSAQGLGDCSIEMGNEYTMIPSQANHAQQQSSTHTGYHQFIPQKRVTEDRT
VNRSGANKCTAGWL
>GhSEP8
MGRVVLKRIENKINRQVTFAKRRNGLLKKAYELSVLCDAEVALIVFSNRGKLYEFCSSPSMTKTLEKYQKCSYSTL
DNSRSISETQQNSYQEYLKLKARVEVLQRSQRNLLGEDLGPLDSKELDQLEHQLEASLKQIRSTKVQAMLDQLH
DLHNREKLLVDANKSLRRKLEELSTQVPQGLAWDNIGGPSIPPYNRLTAAQSEAFFHPLGANCSSQIGYSNDVVS
DEMNAAVHSQNVNGYFPGWML
>GhAG9
MCFSKKIVNGTIAFIMVYFFQLGSMVYPNESLEDSPQKKMGRGKIEIKRIENTTNRQVTFCKRRNGLLKKAYELS
VLCDAEVALIVFSNRGRLYEYANNSVKATIERYKKASDSSNTGSVAEVNAQFYQQEADKLRNQIRNLQNTNRHM
LGESVGGLPMKELKSLESRLEKGISRIRSKKNELLFAEIEYMQKKEIDLHNNNQLLRAKIAENERKQQSMNLMPG
GSSNNFEAIHSQPYDSRNYFQVDALQPAANYYNPQQQQDQIVLQLV
>GhAG10
MILLPSSWFFFWFFGLFQLGSMVYPNESLEDSPQKKMGRGKIEIKRIENTTNRQVTFCKRRNGLLKKAYELSVLC
DAEVALIVFSSRGRLYEYANNSVKATIERYKKASDSSNTGSVAEVNAQFYQQEADKLRNQIRNLQNANRHMLGE
SIGGLPMKELKSLESRLEKGISRIRSKKNELLFAEIEYMQKREIDLHNNNQLLRAKIAENDRKQQSMNLMPGGSS
ANFEALHSQPYDSRNYFQVDALQPATNYYNPQQQQDQIALQLV
>GhTM8.2
MGRGKVELKRIENPTNRQVTFSKRRNGLLKKAFELSILCDAEVALLIFSSSGKVYQFASHDMDRTVAKYRREVGL
PDSSNPQFRTREFWRSEIDELKRSINTSEARLKHLSGEDILALGMRDLKQLERQLKIGVERVRSRKRRIVSDHATLL
KRRHKQLQEENSRLHKRLKELQDGNISSGLVGENACTMFHQSIVHEEDFHNETGLPL
>GhSOC1.7
MVRGKIQIKRIENATSRQVTFSKRRNGLLKKAYELYVLCDAEVAVIIFSHKGKLYEFSSCDNMQNTIERYRQYKKD
VQSNTPQIERYTQQLRLEAENMAKKIEFLEVSKRRMLGQNLGSCSIDELQEVENQLERSLRNIRARKGYLFKEQIL
QLKAKERYMQEENAKLSAKNNGTTCSQQNAEVETELFLGLPENRCS
>GhAGL17.9
MGRGKIAIRRIDNSASRQVTFSKRRKGLIKKAKELAILCDAEVGLVIFSSSGKLYEFASTSMKSVIERYNTLKEEHQQ
LSNPSSEVKFWQREAAILKQQLQNLQDNHRQLMGEQLYGLRVEDLQNLENQLEMSLRGVRMKKERILTNEIEE
LNRRGNLIHQENVELFKKVNLIRKENMELLKKDNKKECSDELRDMLLQFIPSKTHVCEDIYIKDRQ
>GhAGL6.7
MGRGRVELKRIENKINRQVTFSKRRNGLLKKAYELSVLCDAEVALIIFSSRGKLYEFGSSGMSKTLERYQRCCFTP
QDNSLERETQNWYQEVTKLKAKYETLQRTQRHLLGEDLGPLNVKELQNLEKQLEGALALARQRKTQIIIEQMED
LRKKERELGDLNKQLKIKLEAEGQNLKTIQGLWSSGAVAETSNFPLHPSHPHPMDCDHEPVLQIGYHNFVQAE
GSSVPKSMAGETNFIHGWVI
>GhSOC1.8
MVRGKTQMKRIENPTSRQVTFSKRRNGLLKKAFELSVLCDVEVALIIFSPRGKPYEFASSSMQETIERYLRHTKD
NRVKPTEQSMQHLKTEAEKMLKKIELLEVSRRKLLGENLGSCTLEGLQQIEQQLERSVTRVRARKAKVFKDQIEK
LKEKEEVLAAENAMLCEKLKCRVCQYGMLPGKGSKEVNENEEANDESSPSSDVETELFIGLPEGRAKRIVQPNS
TD
>GhAGL15.5
MGRGKIEIKKIENLNSRQVTFSKRRNGLLKKAKELSILCDAEVGVIIFSSTGKVYQWSSTSMEHTLSRYNKGIEED
HSQEHPFDEQQAEKLQGIEVNTMKQEYLRLHTEYMRLNGKELDDLSFKELKQLEDQLNEGIASVERQKEQILM

EQLKRSRLQEQKTIMENEDLRKQVEELRQKGSSNILELNPLLERRLDHSPNNSKADDNNSASDDDNHLSDTSLH
LGLTSNIGRKRKATEIEPTTNDSGSQVASE
>GhSVP7
MTRQRIEIKKIANTAARQVTFSKRRRGLFKKAHELSTLCDAEIALIVFSATGKLFKYSSTSMRQVIERHRLQSERID
GLEGAPSVELQLESATHSVLSKEIAEKTQELRQLRGEDLHGLNLDQLKQLEKLVQGGLSQITETKDERFLKEISTLE
KKGAELKEENLILKQQVENLPLVVKGQPSEPFPHLHKSGDPPPPPQGYNTSDISLTLGLKITI
>GhAGL17.10
MGRGKIVIRRIDNSTSRQVTFSKRRNGLLKKARELSILCDAEVGLIIFSSTGKLYDYASSSMKSVIERYNKVKEEHH
QLLNPDSQVKFWQREAASLRQQLQYLQDYHRQLMGEELSGLSVKDLQHLENQLEVSLKGVRTKKEQILTDEIKE
LNHKGHLIHQENLELYKKVDLIHQQNTELQKKIYGTREANEASRISHSNYTFNNGYDLHAPVRLQLSQPQPPKN
NAPAKSMKLG
>GhAP3.9
MGRGKIEIKRIENATNRQVTYSKRRNGIFKKAQELTVLCDAKVSLIMFSSTGKFHEFISPNISTKAFFDLYQKTTGT
DLWISHYEKMQENYRRLKEINKKSRREISRQRMGGDLDDLNIKELQALEAKMDSSLVAIRDRKYHVIKTQTDTH
RKKVSESNTIKTVAFHSISRVLIIFEDWNALQVRNLEERHANLVFDLLSWEEQSGFGGFDNEIFHGDVDKSGTFV
SQPRQVHIIQPLSGAEPDSDSPKTTPPISVYISDISTLLLLAELYSFSFQLKALGHKIKILCKSVPPLVKLKSGDECGRSI
FTPVAHVLSTNSCICKNTTDVDTQNGLVDDGLYDNCDCVEVVREDGSSREIWAEYLLVEVIFAT
>GhSVP8
MAREKIQIKKIDNSTARQVTFAKRRRGLFKKAEELAILCDADVALIIFSSTGKLFDYASSSMKEILERHHLRSKNLEK
LEQPCLGLQLLEHSNQSMLTMEIAEKSHQLRQMRGEELHGLNIEELQQLEKSLEIGLSRVMEKKGQRIMREIKD
LQRKGMQLMEENERLKQQIINGPRQVAGDSDNIIFGEEGQSSESVTNVCTSNGNPHDYESSVTSLKLG
>GhAGL15.6
MGRGKIEIKRIENANSRQVTFSKRRAGLLKKAKELAILCDAEVAVIIFSNTGKLFEFSSSGMNKTISRYKSAQGSPEI
AQVEHKAEKQDSKEADHLKDEIAKLQMKQLQLLGKNLTSMSLKELQLLEQQLNEGLLSVKEKKEQLLMQQLEQ
SRLQEQRAMLENETLRRQASYVEELRGFFPTTDHPIQPYLECYPVERKNSLMSHSIPSPDLTCNCTVEKGDSDTT
LYLGLPSDYHKRKKPEIESHSNESESQLGLL
>GhSOC1.9
MCFVGFEGTKILIMVRGKTQMRRIENDTSRQVTFSKRRNGLLKKAFELSVLCDAEVALIIFSPRGKLFEFASSSMQ
QTIERYRRHTKDNETNKPIEQNLQHLKTESANMLKTLEDLEVSRRKLLGENLGSCTLEELQEIEQQLEKSVSIIRAR
KLIANNYLIIFLAQEKALAAENEKLFFLVKLFMSQCGTKSWKGLSEQEDNVPYDESSPSSDVETKLFIGLPEGRTRR
IAQLN
>GhSOC1.10
MVRRRTQMKRIENAASRQVTFSKRRNGLLKKAFELSVLCDAEVALIIFSPRGKLYEFSSSSTNKTIERYQKRQKDI
HGISSKGEDMQDDVKEDAHSLAKKIESLEDSKRKLLGHGLEPCSIDDLLLLEKQLERSLSRIRARKNQVFTEQIKKL
KEEERRLGEENANLREECGMRPRESTSTRQSDDERNMEVETELCIGPPERRCKLKP
>GhAGL17.11
MGRGKIEIKKIEKSSSRQVTFSKRRNGLLKKAKELAILCDAEVGLIIFSSTSKLHHFASSSMNSVIERYNKYKEENHH
QLLDPASELKFWKKEVASLRQQLNDLQECQRQLMGKELSGLSFKDLQGLENQLQMSLKRVRMRKDQILTNQI
DELNRKGHHIHQENLEVHKKLDLICHENTELQKKVNGNGTEEANEGSKSLSHSYGFNNGYDYLQAPVVDLRLS
QPQQLPDADTSNLKTR
>GhSVP9
MTRQKIQIKKIDNTAARQVTFSKRRRGLFKKAYELSTLCDAEIALLVFSNTGKLFEYSSTSTRQVIERRNLQSERIDL
LDPISTLELQLQSSTCAMLGTEIAEKTKELRQLRGEELQGLDLEELKHLEKLLEGGLNRVTQTKDELFFKEISILKRKE
VELMEENQQLKEKMGNSPHVVQPTVAQQGLGQPSDCNGHAWRSYSSDISLRLGLPYPN

>GhBS4
MGRGKIAIKRIENQTTRQVTFSKRRAGLLKKTHELSVLCDAQIGLIIFSSTGKMCQYCTQPYRMEQIIERYQKVTG
TRIPEHDNREHLYNELAVLRKETRRLQLSMRRYTGEDMSSIPYRSVNKVRERKNELLQQQLDNLRRKERMLEEE
NNNMYRWIQEHRAAIEYQQHGGLEAKPVEHHQQVLDEFPFYGEPSSVLQLATIPQQFSYQLQLAQPNLQDSN
V
>GhSEP9
MGRGRVELKRIENKINRQVTFAKRRNGLLKKAYELSILCDAEVALIIFSNRGKLYEFCSTSSMAKTLEKYNSYTYGA
LEPGQTEIDAQSNYQEYLKLKSKVEVLQQSQRHFLGEEIADLGTKELEQLEHQLDFSLKKIRSTKMQLMIDQLSEL
QTKEEVLLETNRNLRMKLDGSGPSMRSSWETGEHSIPYNHPPPPPQSEGFFEPLHCNNSLQIGYNPISVTVEDT
ATASALAPSGFIPGWML
>GhAPI.11
MGRGRVQLKRIENKINRQVTFSKRRAGLLKKAHEISILCDAEVALIVFSHKGKLFEYSTDSCMEKILERYERYSYAE
RQLVATEPESQGNWSMDYNRLKAKVELLQRNHRHYMGEELESLSLKELQNLEQQLDTALKLIRSKKNQLMYESI
SELQRKEKAIQEQNTMLAKQIKEREKTVAQQQQQQQQPQWGQQNHGLNTSSFLLPQPPPCLNIGGTYQEE
TTEMRRNELDLTLEPIYSCHLGCFAA
>GhAGL12.2
MGRGRVQMKRIENPVHRQVTFCKRRAGLLKKAKELSVLCDAEIGVVIFSAHGKLYELATKGTMQELIERYGKYT
GGPPADEPMVEPMDAKKEIEMLKQEIEILQKGPRYMFGGGCEYMSLNELLVLEKHLEIWINHIRSTIMDIMFQE
IQMLRNKEGIMTAANKCLHETIEENISYTDFTTMTTNIMHPLTIANNIF
>GhAGL17.12
MGRGKIVIRRIDNSTSRQVTFSKRRNGLLKKAKELAILCDAEVGVMIFSSTGKLYDFASTSMKSVIERYNKTKEEH
QQPENPTSEVKFWQREAAVLRQQLQSLQENHRQMMGEELSGLTVKDLQNLESQLEMSLRGVRMKKDQML
MDEIQELTRKGNLIHQENVELYKKVNQIRQENTELYKKVYGTRDVNGANKELVVTNGPSIGEDIHVPVHLQLSQ
PQQQNYETPTRATKLGSPSIAKQILKDHNAIFASCDIPMAAINGTFGGLDFVWRSNNPKLHKLRKLVICEIMINN
GLNACYELCR
>GhAP3.10
MGRGKIEIKRIENSSNRQVTYSKRRNGIMKKAKEITILCDAKVSLIIFGSSGKMHEYCSPSTNLVDILDQYQKTSGK
KLWDAKHEAISLKAQNNNGCNRSQCKIYLVHDGSSRNLSNEIDRIKKENDNMQIELRHLKGEDITSLPYKELMAI
EDALENGLTYVRGKQDIKAYQSLFSFSFVQMDVLDKTWKNTKFLEEDYKQLSFILNQQQVAYESAREQMDHGY
QRARDYNSQMSSTFQVQPMQPNLQERM
>GhMIKC*2
MGRVKLKIKKLENPNGRQATYAKRKHGIMKKANELSILCDVEIVLLMFSPTNKPSLCIGKRSSIEEIIEKFAQLTPQE
RAKRYAQIFCALKKTFKKLDHDVNIHEFLGSSTQTIEELTNQARLLQTRLSEVHRRLSCWTNVDKINNVEQLGQM
EDSLKEYLNQIQAHKENLGKQQQLLSLECTSQFQNEMQVPYRMGLEHQLQTLSWLPNQDSRHIALSDDPNLIP
HRDVECSASSSFGSYSGYFGTGKSSEPSSSGQENSILNDLSGNGSLQLQLGGQCPLFSYNLNILNDPKFPPVAEM
NFNETPADYQVNGALQGPRTGFDTAPGSWASTSGSCAVTMFDEPLYTRLQQH
>GhMIKC*11
MGRVKLKIKKLENPNGRQATYTKRKHGIMKKANELSILCDVEIVLLMFSPTNKPSLCIGKRSSLEEIIEKFAQLTPQ
ERAKSRKLESLEALKKTFKKLDHDVNIHEFLGSSTQTIEDLTNQARLLQTRLSEVHRRLSCWTNVDKINNVEQLG
QMEDSLKEYLNQIQSHKACHFPKYVMTKYYLTSLFLQFQNEMQVPYRMGLEQQLQTLSWLPNQDSRHIALSD
DPNLIPHRDVECSASSSFGSYSGYFGTGKSSELSSSGQENSILNDLSGNGSLQLQLGGQCPLFSYNLNILNDPKFP
PVAEMNFNETPADYQVNGALQGPRTGFDTTPGSWPSTSGSCAVTMFDEPLYTRLQQH
>GhMIKC*8
MGRVKLKIKKLENTNGRQATYAKRKHGIMKKANELSILCDVEIILLMFSPTNKPSVCIGKRSSIEEILEKFAQLTPQE

RAKRCSQNFQALKKTFKKLDHDVNIHEFLGSRYMTIFFLYICNCYGQDLTNQARLLQARLSEIHRRLSCWTDVDK
INNVEHLGQMEDSLKDYLNQIRAHKENLGKQQLLPIECTSQFQNEMHVPFRMGIEQQLQSLAWMPNNDSRH
MALTEDPNLIPPRDVECSASSSFGSYSGYFGTPKSSELSSSGQENGILNDLQLGGQCPFFSYDLSILNDHKFQPVA
EMNFPETPVDYHVNGVLAGPRAGYDPNQGSWASTSGPCAVTMFDEPLYTGQLN
>GhMIKC*18
MGRVKLKIKKLENTNGRQATYAKRKHGIMKKANELSILCDVEIILLMFSPTNKPSVCIGKRSSIEEIIEKFAQLTPQE
RAKRCSQIFQALKKTFKKLDHDVNIHEFLGSRYMTIFILYICNCYGQDLTNQARLLQARLSEIHRRLSCWTDVDKI
NNVEHLGQMEDSLKDYLNQIRAHKENLGKQQLLPIECTSQFQNEMHVPFRMGIEQQLQSLAWMPNNDSRH
MALPEDPNLIPPRDVECSASSSFGSYSGYFGTPKSSELSSSGQENGILNDLQLGGQCPFFSYDLSILNDRKFPPVA
EMNFPETPVDYHVNGVLAGPRAGYDPNQGSWASTSGPCAVTMFDEPLYTGAAIHITCWHGKGLIGEYSQDK
KTLDVLTASMDKIARNLSRETSPQLSWI
>GhMIKC*17
MGRVKLKIKRLESYSNRQVTYSKRRTGILKKAKELSILCDIHIILLMFSPTGKPTLFHGERSTIEEVIAKFAQLTPQER
AKRKLESLEALKKTFKKLDHDLNIQDFLGATQSVEEMTNEVSMLQARLNEVHKRLSYWNNPDKIDNIEHLRQM
ENSLRESIERIRIHKENYGKHHLLPLESTSQFQNAMPLPVMIGGVQEAQPVTWLPNNGNQQMLLHNESNFLP
NLDTECATDGSLAGYSGFFVPGKQTDIGNSVQVDNTIQESNVLNDLGNNAFLNSQLGNLQGDPRVYQVITDFE
APRPMSNGGHQAWISSSGPCGIAMFDGNSYHQELNLPAKSLIQIVVYRSKQNRLL
>GhMIKC*15
MGRVKLKIKRLESYSNRQVTYSKRRTGILKKAKELSILCDIHIILLMFSPTGKPTLFHGERSNIEEVIVKFAQLTPQER
AKRKLESLEALKKTFMKLDHDLNIHDFLGAKYEMTKEVSRFRAQLAEVHKRLSYWSNPDKIDNIEHLRQMEDSL
RESIERVRIHKENFGKHHLMSLECCNQFQNRIPLSVMIGAVQEAQPVMWLPNNENHHTLLHNELNFLPHRDA
ECSTDCSLAGYSGFFGSGKQTEISSSGQVDNVVQECNALNELGSNACLNLEPGEQYFYQPYSASNYQDDEKLKT
EMEVNLQGNPVVNQVISNFEIPRPMYNNGHQAWVLSSGPCGIAMFDGNSYHQFLMMNTL
>GhMIKC*6
MGRVKLKIKRLESYSNRQVTYSKRRTGILKKAKELSILCDIHIILLMFSPTGKPTLFHGERSNIEEVIVKFAQLTPQER
AKRKLESLEALKKTFMKLDHDLNIHDFLGAKYEMTKEVSRFRAQLAEVHKRLSYWSNPDKIDNIEHLRQMEDSL
RESIERVRIHKENFGKHHLMSLECCNQVAKIFSPPVCVCFWERYLTTYDGLQFQNRIPLSVMIGAVQEAQPVM
WLPNNENHHMLLHNELNFLPHRDAECSTDCSLAGYSGFFGSGKQTEISSSGQVDNVVQECNALNELGSNACL
NLEPGEQYFYQPYSASNYQDDEKLKTEMEVNLQGNPVVNQVISNFEIPRPMYNNGHQAWVLSSGPCGIAMF
DGNSYHQVRII
>GhMIKC*7
MGRVKLKIKRLESYSNRQVTYSKRRTGILKKAKELSILCDIHIILLMFSPTGKPTLFHGERSTIEEVIAKFAQLTPQER
AKRKLESLEALKKTFKKLDHDLNIQDFLGATQSVEEMTNEVSMLQARLNEVNKRLSYWNNPDKIDNIEHLRQM
EDSLRESIERIRIHKENYGKHHLLPLESTSQFQNAMPLPVMIGGVQEAQPVTWLPNNDNQQMLLHNESNFLP
NLDTECPTDGSLAGYSGFFVPGKQTDIGNSVQVDNTIQESNVLNDLGNNAFLNSQLGKQYLYPQFSASNLQDD
EKLKSEMVGNLQGGPGVYQVITDFEAPRTMSNGGHQAWITSSGPCGIAMFDGNSYHQELNLPAKSLIQIVVY
RSKQNRLL
>GhMIKC*4
MGRKKLKIQRLEDLKARQAKYSKRKTGILKKAKELNILCEVDVALLFSSPSGRPTLFVGQNSTGLSSILKRLSNLSFE
EREERRAYTIEMLKKIYENSESEFDLLSLPHATNADTLKLYEDELQELKDKLVEKSKILRDWRNPNNVEDLNQIKM
MEDHLIASLNGLRSRRNQLAMEQQIRERELEGNENLET
>GhMIKC*3
MGRVKLQIKRIENTTNRQVTFSKRRNGLIKKAYELSVLCDVDVALIMFSPSGRLSLFSGNKSIEEILGRYVNLPEHE
RGRLRNKEFLIKALGKLRNEADQTSQPTSPESINPQLEEFQQEIINCKSRIANMEKRLRIFEGDASEITTLIQADYH

EQILEETLKQVRLHKQVLEEKYTSPGPPPTTKVKLPPNAGDVNGFVTASSSSISNHFLNFLDSNGLLPPGCASIIDE
APAIAEILPPHSTDLLNEEEINVEEHLSLTRGLENHNKVQHPEFGQVNDVMFPWTQLYPTGSTWGPNKLVRGW
AFEA
>GhMIKC*14
MGRRKLKIQRLEDMKARQAKYSKRKKGILKKAKELSILCDVEIVLLLSSPSGKPTLFVGQNPNGLYCILQKVSNMP
FVEREERRAYTLEMLKKFYVNWESKFDPLSLPRNNNVDTLKLYEDQLQELKDKLTKKSKILRDWKYPENVEDLN
QIKFMEDHLIASLNGLRNRKNQLAMEQQSKERYLEVGL
>GhMIKC*13
MGRVKLQIKRIENTTNRQVTFSKRRNGLIKKAYELSVLCDVDVALIMFSPSGRLSLFSGNKSIEEILGRYVNLPEHE
RGRLRNKEFPIKALGKLRDEADQTSQPTSSPECINPQLEEFQQEIINCKSRIANMEKRLRIFEGDASEITTLIQADY
HEQILEETLKQVRLHKQVLEEKFTSPGPPPTTKGKLPPEAGDVNGFVTTSSSSISNHFLNFLDSNGLLPPGCASIID
EAPAVAEILPPQSTDLLNEEEINVEEQLSLARGLENHNNVQRPEFGQVIDVNLSPWTQLYPKGSIWGPNKLVHG
WAFEA
>GhMIKC*10
MGRVKLQIKRIENTTNRQVTFSKRRNGLIKKAYELSVLCDVDVALIMFSPSGRLSLFSRNKSIEEILERYVNLPEHE
RGSLLKIDAYIHTYFQFLLKALGKLRDEADQIQTYQAASPESTDSQLEELQQEIVKCKSRIADIQRRLRIFEGEEITTL
SQAEFHKQILEETLQQVRLRKQGLQEKATSSSSALDWIRKKDPQVHILNFLESNGLLPQRDECQSVAENILPPLD
GEEIINVGDQLSPTRSGLDNSNNMQRTELGQVNNVNLSPWTELYSTVAGNDSFPDGQPAAGGGGRALLELYV
SQFTQSAISTMNQHHT
>GhMIKC*5
MGRRKLKIQRLEDMKARQAKYSKRKKGILKKAKELSILCDVEVVLLLSSPSGKRTFFVGQDPNCLYNILQKVSNM
PFVEREERRAYTMEMLKKFYVNWESEFDPLSPPRNKNVDILKLYEDQVQELKDKLTKKSKILRDWKNPENVKDL
NQIKFMEDHLIASLNGLRNRKNQLAMEQQSRQRYLEVGL
>GhMIKC*9
MGRVKLEIKRIENNTNRQVTFSKRRNGLIKKAYELSILCDIDIALIMFSPSGRISHFSGRRRIEDVFMRYINLPDRER
EHDIQNKEYMLRILQQLRSENDVALQLANPASFNSDFKEIQQEIVRLQQQLQIAEDQLRAYEPDPFRFTSMAEL
ESCEKHLVETLANVVQRKEYILSNHLSSYDPSPIQQGLPPSFENEVVNWLPDNGQNQSQIFDASASLNPLRDLSS
TVYDPLLQGSMSNVDPHNIGDQCHVSNPNTENFAPWPRSFASTGLQSNSMPPTLYSHVQQHGMVSHQEMA
EMVPNDQQMEIPGNYNSHGQMADNEGSNYENRVHEHNGQ
>GhMIKC*12
MGRKKLKIQRLEDLKARQAKYSKRKIGILKKAKELNILCEVDVALLFSSPSGRPTLFVGKNSKGLSSILKRLSNLSFE
EREERRAYTIEMLKKIYENSESEFDPLSLSYDTNADTLKLYKDELQELKHILVEKSKILRDWRNPNNVEDLNQIKM
MEDHLIASINGLRSRKNQLAMEQQIRERESEGNENLET
>GhMIKC*1
MGRVKLEIKRIENNTNRQVTFSKRRNGLIKKAYELSILCDIDIALIMFSPSGRISHFSGRRRIEDVFMRYINLPDRER
EHDIQNKEYMLRILQQLRSENDIALQLAKFEIQQEIVRLQQQLQMAEDQLRAYEPDPFRFTSMAELESCEKHLV
ETLANVVQRKEYILSNHLSSYDPSPIQQGLPPSFENEVVNWLPDNGQNQSQIFDASASLNPLRDLSSTVYDPLL
QGSMSNVDPHNIGDQCHVSNPNTENFAPWPQSFASTGLQSNSMPPTLYSHVQQHGMVDHQEMAEMVPS
DQQMEIPGNYNSHGQMADNEGSNYENRVHEHNGQ
>GhMIKC*16
MGRVKLQIKRIENDTNRQVTFSKRRNGLIKKAYELSILCDIEIALIMFSPSGRVSHFSGKKRIEDVLSRYINLPDQDR
GCTLKKLQAEHDFVLQLASSTTNTTTNSNVEELHREINNLRQQIQLAEEQLRVYEPEPEPLTLTSMAEFESCEKNL
EQVLTRITQRKNYLTTNHLSSFTDPSSVQMYLDAQEGMPNSFGNDVVGWLPDNGQSQNPTQYCAGSESPSIP
VRNQSSMNTVYDPMGHGTNMSEMGGCHVTTTSSNDGLPSWHHNYSSTELLSAFMSSPTSFPLIKDIGDPSIP

TKQEQVESGTNFPQMPCRDEGASKLPHLNVD
